# Distinct effect of calorie restriction between congenic mating types of *Cryptococcus neoformans*

**DOI:** 10.1038/s41598-024-69087-y

**Published:** 2024-08-06

**Authors:** Natalia Kronbauer Oliveira, Kyungyoon Yoo, Somanon Bhattacharya, Rina Gambhir, Nigina Kirgizbaeva, Pilar Arcones García, Ignacio Pompa Prados, Caroline Mota Fernandes, Maurizio Del Poeta, Bettina C. Fries

**Affiliations:** 1 Department of Microbiology and Immunology, Renaissance School of Medicine, Stony Brook University, Stony Brook, NY 11794 USA; 2 Division of Infectious Diseases, Department of Medicine, Stony Brook University, Stony Brook, NY 11794 USA; 3 Stony Brook University, Stony Brook, NY 11794 USA; 4 Veterans Administration Medical Center, Northport, NY 11768 USA

**Keywords:** Fungal pathogenesis, Microbiology, Fungi, Fungal biology

## Abstract

*Cryptococcus neoformans* (*Cn*) is an opportunistic yeast that causes meningoencephalitis in immunocompromised individuals. Calorie restriction (CR) prolongs *Cn* replicative lifespan (RLS) and mimics low-glucose environments in which *Cn* resides during infection. The effects of CR-mediated stress can differ among strains and have only been studied in MATα cells. *Cn* replicates sexually, generating two mating types, MATα and MAT**a**. MATα strains are more dominant in clinical and environmental isolates. We sought to compare the effects of CR stress and longevity regulation between congenic MATα and MAT**a**. Although MATα and MAT**a** cells extended their RLS in response to CR, they engaged different pathways. The sirtuins were upregulated in MATα cells under CR, but not in MAT**a** cells. RLS extension was *SIR2*-dependent in KN99α, but not in KN99**a**. The TOR nutrient-sensing pathway was downregulated in MAT**a** strains under CR, while MATα strains demonstrated no difference. Lower oxidative stress and higher ATP production were observed in KN99α cells, possibly due to higher SOD expression. *SIR2* was important for mitochondrial morphology and function in both mating types. Increased ATP production during CR powered the upregulated ABC transporters, increasing efflux in MATα cells. This led to enhanced fluconazole tolerance, while MAT**a** cells remained sensitive to fluconazole. Our investigation highlights differences in the response of the mating types to CR.

## Introduction

*Cryptococcus neoformans* (*Cn*) is an environmental pathogenic fungus that most commonly affects immunocompromised individuals, such as AIDS patients. Upon inhalation, the fungus causes a lung infection and eventually disseminates to the brain, causing life-threatening meningoencephalitis. An estimated 180,000 deaths occur annually despite antifungal therapy^1^.

*Cn* exhibits a bipolar mating system with two distinct mating types, MATα and MAT**a**^2–5^. Interestingly*,* the MATα type is 30–40 times more prevalent than MAT**a** type among clinical and environmental isolates^6^. Congenic MAT**a**/MATα mating pairs, which are genetically identical except for their mating locus, have been created through repeated backcrossing events. Studies using these strains have shown that MATα cells exhibit enhanced virulence in certain genetic backgrounds^7–10^ and possess a competitive advantage over their respective congenic MAT**a** cells in brain colonization^11^. However, the underlying reasons for the higher prevalence of MATα cells are still unclear.

At the molecular level, the *MAT* locus spans over 100 kb and contains more than 20 genes that bear non-identical alleles rearranged differently in the two mating loci. Importantly, these genes influence mating type determination and various virulence traits, including melanin production, capsule formation, and the ability to thrive at elevated temperatures (37 °C)^9,12–14^. The *MAT* locus also harbors transcription factors, such as *ZNF1*α/**a**, *STE12*α/**a**, and *SXI1*α/2**a**, which orchestrate downstream responses to nutritional cues like glucose starvation^15–17^.

In the human host, *Cn* reproduces asexually in brain and lung tissues, which are characterized by low glucose levels. This process involves asymmetric cell divisions, resulting in progressive phenotypic changes in aging mother cells compared to their daughter cells. These changes include increased resistance to phagocytosis, macrophage-mediated killing, and antifungal drugs, ultimately contributing to the resilience and accumulation of old *Cn* cells within the host^18–20^. Calorie restriction (CR), which reduces glucose availability, closely mimics the low-glucose environments encountered by *Cn* cells during chronic infections^21^. CR can influence the replicative lifespan (RLS) of various organisms by modulating several pathways and also impacts antifungal resistance to azoles^22^.

Sir2, a member of the sirtuin family of highly conserved NAD+-dependent histone deacetylases, is associated with increased RLS in multiple organisms^23–25^. Early studies demonstrated that reducing glucose availability fails to increase the lifespan in *SIR2* deletion mutants of *Saccharomyces cerevisiae* (*Sc*) and *Cn,* suggesting that lifespan extension during CR was mediated by Sir2 activation^26,27^. Similarly, reduced signaling of the nutrient-responsive TOR and PKA pathways can promote CR-mediated RLS extension^28^. TOR reduction regulates the phosphorylation of transcription factors involved in mitochondrial function and stress tolerance^29^. CR increases mitochondrial biogenesis and respiration, decreasing oxidative damage, which is crucial as mitochondrial dysfunction is a hallmark of aging^30,31^. Both the TOR pathway and the sirtuins are associated with the maintenance of mitochondrial function and morphology^32,33^.

In this study, we hypothesize that distinct regulatory mechanisms governing longevity in *Cn* mating types are influenced by low glucose stress conditions. We propose that these different stress responses to nutrient deprivation may explain the observed differences in prevalence rates between MATα and MAT**a** types. To test this hypothesis, we assessed the expression levels of key longevity pathways in both MATα and MAT**a** cells of serotypes A and D. Additionally, we examined mitochondrial function and antifungal tolerance in MATα and MAT**a** cells. Our findings indicate that CR exerts a variable pro-longevity effect on *Cn* mating types, with differential involvement of the *SIR2* and TOR pathways. This ultimately impacts mitochondrial function and antifungal drug resistance in a mating type-dependent manner.

## Results

### Calorie restriction extends lifespan of both mating types

In the human host, *Cn* experiences low glucose environments, especially in lung and brain tissues. Given the notable disparity between the prevalence of MAT**a** and MATα cells during human infections, we investigated the impact of CR on the RLS of both mating types in two strains: KN99 (serotype A) and JEC (serotype D) in both standard synthetic media (SM) and calorie-restricted (CR) conditions.

**Figure 1:**
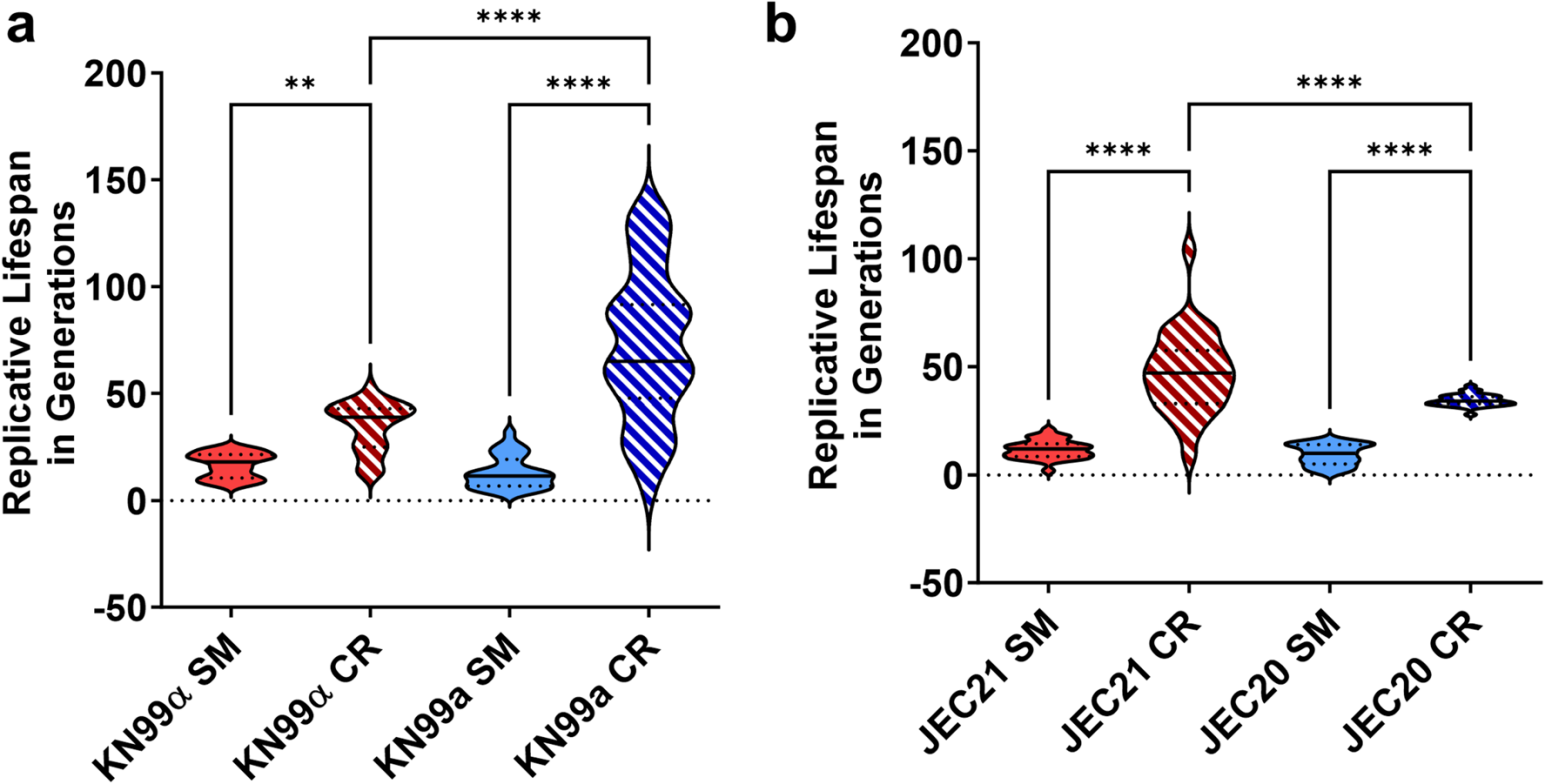
CR Extends RLS in *Cn* Strains. Calorie restriction media (0.05% glucose, CR) extended the replicative lifespan (RLS) of two *Cn* mating pairs (**a**) KN99α (MATα; *p* < 0.01) and KN99**a** (MAT**a;** *p* < 0.0001), (**b**) JEC21 (MATα; *p* < 0.0001) and JEC20 (MAT**a**; *p* < 0.0001) compared to synthetic media (2% glucose, SM) as analyzed by microdissection. Statistical analysis was performed with One-Way ANOVA.

Under standard conditions, the RLS for KN99α and KN99**a** was comparable (16.5 vs. 13.1 generations) (Fig. 1a). However, CR had a more pronounced pro-longevity effect on KN99**a**, boosting its mean RLS by a striking 451% (13.1 to 72.3 generations), whereas the RLS increased by 109% in KN99α cells grown under CR (16.5 to 34.6 generations). Similarly, JEC21 (MATα) and JEC20 (MAT**a**) had comparable RLS (11.7 vs 9.3 generations) (Fig. 1b). When subjected to CR, the JEC21 mean RLS saw a 300% increase (11.7 to 47.2 generations), whereas JEC20 saw a RLS extension of 270% with CR (9.3 to 34.4 generations). In KN99α, KN99**a**, and JEC21, CR also significantly increased the variability of the RLS (stochasticity) when compared to the same strain grown under SM media. These findings demonstrate CR had a significant pro-longevity effect on both mating types in two different *Cn* strains.

### Effect of Sir2 on lifespan extension in MATα and MATa

**Figure 2:**
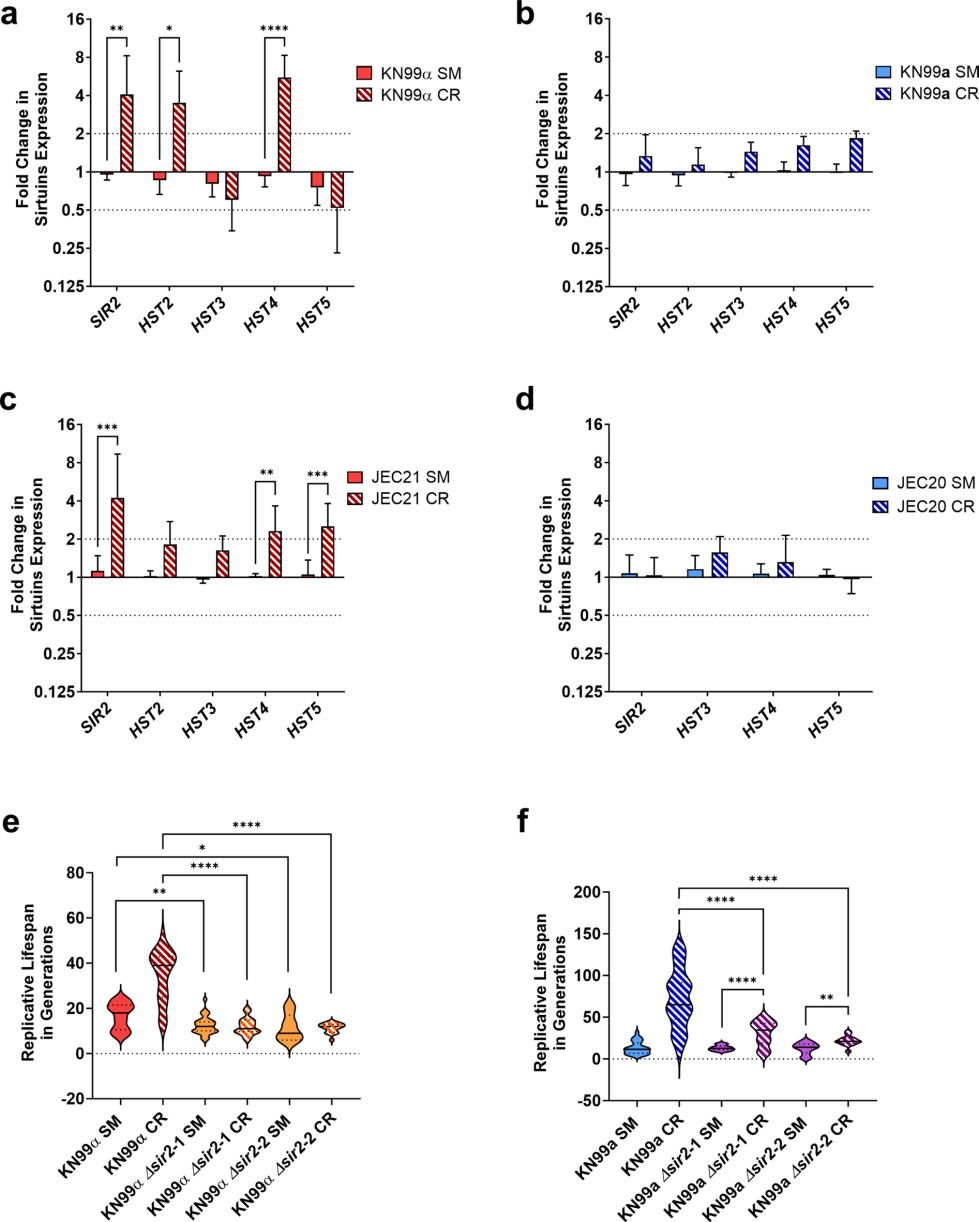
Pro-longevity Effect is *SIR2*-dependent in KN99α. CR led to the upregulation of (**a**) *SIR2* (4.07-fold, *p* = 0.0023), *HST2* (3.4-fold, *p* = 0.014), and *HST4* (5.5-fold, *p* < 0.0001) in KN99α compared to SM media (MATα), while (**b**) KN99**a** showed no statistical difference in expression (MAT**a**). Similarly, CR led to the upregulation of (**c**) *SIR2* (4.2-fold, *p* = 0.0002), *HST4* (2.3-fold, *p* = 0.0029), and *HST5* (2.5-fold, *p* = 0.0008) in JEC21 compared to SM media (MATα) while (**d**) JEC20 showed no statistical difference in expression (MAT**a**). qPCR was used to analyze expression and *ACT1* was used as an internal control. The dotted lines signify a twofold up- or downregulation of the genes. qPCR was performed in biological triplicate and error bars signify standard deviation between samples. (**e**) Deletion of *SIR2* in KN99α (KN99α *Δsir2*-1 and KN99α *Δsir2*-2) shortened RLS compared to the parental strain (*p* = 0.0024 and *p* = 0.028). CR-induced pro-longevity effect of KN99α was lost on the mutant strain (KN99α *Δsir2* CR; *p* < 0.0001). (**f**) Deletion of *SIR2* in KN99**a** (KN99**a** *Δsir2*-1 and KN99**a** *Δsir2*-2) had no change in RLS compared to the parental strain. CR extended the RLS of the mutant strains (*p* < 0.0001 and *p* = 0.0022) but to smaller extents than the parental strain. Statistical analysis was performed with Two-Way ANOVA and Student’s *t*-test with Welch’s correction.

The highly conserved family of NAD+-dependent histone deacetylase, known as sirtuins, modulate RLS in several organisms^23–25^. Recognizing the already established role of *SIR2* in CR-mediated longevity of the H99 strain^27^, we compared the transcriptional regulation of the sirtuin family under calorie-restricted growth conditions in both mating types. Notably, *SIR2* was similarly upregulated fourfold in KN99α (Fig. 2a) and JEC21 (Fig. 2c) when subjected to CR. Furthermore, CR led to the upregulation of other sirtuins in MATα strains. In KN99α, mRNA levels of *HST2* and *HST4* were elevated (3.5-fold and 5.5-fold, respectively), whereas in JEC21, *HST4* and *HST5* mRNA levels were elevated (2.3-fold and 2.5-fold, respectively). In contrast, sirtuins were not regulated in the MAT**a** strains KN99**a** and JEC20 during CR (Fig. 2b and d). Collectively, these findings suggest that under CR, sirtuin regulation occurs in MATα strains, but not in MAT**a** strains.

To further explore the impact of *SIR2* on RLS extension, we employed *Δsir2* mutants of both KN99α and KN99**a**. A KN99α *Δsir2*-1 strain from the Madhani knockout collection was used. A second KN99α *Δsir2*-2 mutant and two KN99**a** *Δsir2* mutants were generated employing a TRACE CRISPR-Cas9 system. Successful deletion was verified by PCR of the complete *SIR2* gene. In addition, *SIR2* mRNA expression by qPCR verified the lack of *SIR2* transcription (Fig. S1). Finally, the whole deletion locus was sequenced to confirm proper integration (Fig. S1 and Supplemental Data: Sequencing). Consistent with previous findings, the removal of the *SIR2* gene in KN99α led to a notable reduction in RLS in both mutant strains (KN99α *Δsir2*-1: 25%, 16.5 to 12.4 generations; KN99α *Δsir2*-2: 30%, 16.5 to 11.6 generations) when compared to the wild-type strain in SM media (Fig. 2e). When placed under CR, both mutants failed to extend their RLS (KN99α *Δsir2*-1: 12; KN99α *Δsir2*-2: 11.3 generations). This observation reinforces that CR-induced extension of RLS is contingent upon the presence of *SIR2* in KN99α. Contrastingly, in the KN99**a** strain, loss of *SIR2* had no effect on the mean RLS compared to the wild-type in SM media (KN99**a** *Δsir2*-1: 13.1 vs 13.1; KN99**a** *Δsir2*-2: 13.1 vs 12.6 generations) (Fig. 2f). CR had a substantial pro-longevity effect on the RLS of both KN99**a** mutants, (KN99**a** *Δsir2*-1: 133%, 13.1 to 30.6; KN99**a** *Δsir2*-2: 71%, 12.6 to 21.6 generations) although to a lesser extent than wild-type. These findings reinforce that *SIR2* is not essential to the pro-longevity effect of CR in KN99**a**, suggesting it might play a secondary role in MAT**a** strains.

The role of the remaining sirtuins on *Cn* lifespan was not known. Analysis of the RLS of *Δhst2*, *Δhst3*, *Δhst4*, and *Δhst5* mutants indicated no change in RLS relative to the wild-type in SM media, therefore RLS determination under CR was not further pursued (Fig. S2). In summary, these data suggest that *SIR2* plays a divergent role in MAT**a** than in MATα when the fungus is grown under CR whereas the other sirtuins do not appear to affect RLS in either mating type.

### TOR and PKA pathway dynamics in MATα and MATa cells

**Figure 3:**
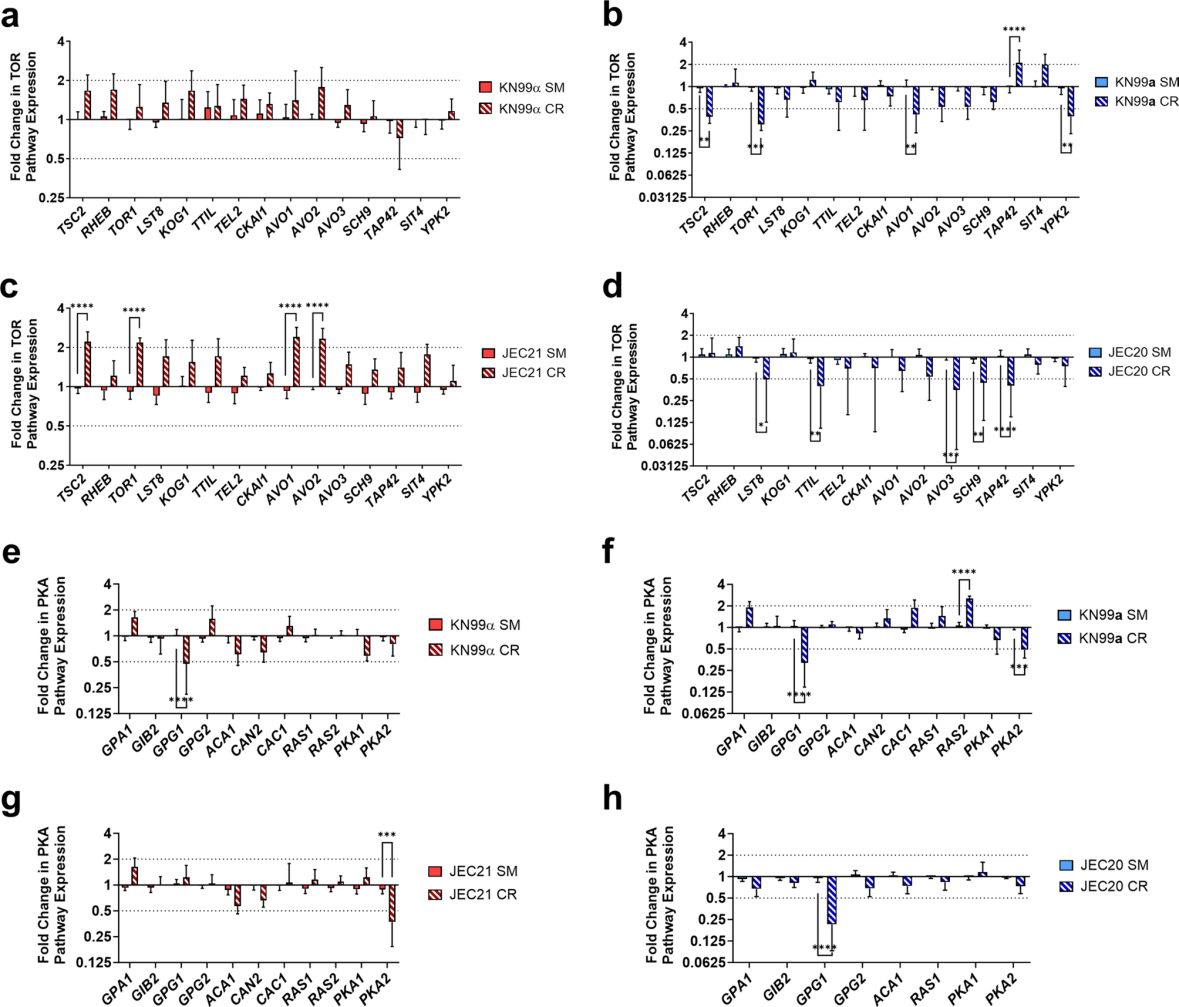
TOR Pathway is Downregulated in MAT**a** cells. (**a**) KN99α (MATα) did not regulate TOR genes under CR compared to SM media, whereas in (**c**) JEC21 (MATα) genes *TSC2* (2.2-fold, *p* < 0.0001), *TOR1* (2.1-fold, *p* < 0.0001), *AVO1* (2.4-fold, *p* < 0.0001), and *AVO2* (2.3-fold, *p* < 0.0001) were upregulated in CR compared to SM media. (**b**) TOR pathway genes *TSC2* (0.38-fold, *p* = 0.0064), *TOR1* (0.3-fold, *p* = 0.0003), *AVO1* (0.41-fold, *p* = 0.0017), and *YPK2* (0.39-fold, *p* = 0.0062) were downregulated in KN99**a** (MAT**a**) during CR compared to SM media, while *TAP42* (2.08-fold, *p* < 0.0001) was upregulated. (**d**) Similarly, JEC20 (MAT**a**) downregulated TOR pathway genes *LST8* (0.49-fold, *p* = 0.013), *TTIL* (0.39-fold, *p* < 0.0013), *AVO3* (0.35-fold, *p* < 0.0002), *SCH9* (0.44-fold, *p* < 0.0054), and *TAP42* (0.4-fold, *p* < 0.0001) during CR compared to SM media. (**e**) PKA pathway gene *GPG1* (0.47-fold, *p* < 0.0001) was downregulated in KN99α during CR compared to SM media. (**f**) PKA pathway genes *GPG1* (0.31-fold, *p* < 0.0001) and *PKA2* (0.48-fold, *p* = 0.0005) were downregulated, while *RAS2* (2.5-fold, *p* < 0.0001) was upregulated in KN99**a** during CR compared to SM media. (**g**) *PKA2* (0.37-fold, *p* = 0.0004) was downregulated in JEC21 in CR growth conditions compared to SM media. (**h**) *GPG1* (0.21-fold, *p* < 0.0001) was downregulated in JEC20 in CR growth conditions compared to SM media. qPCR was used to analyze expression and *ACT1* was used as an internal control. The dotted lines signify a twofold up- or downregulation of the genes. qPCR was performed in biological triplicate and error bars signify standard deviation between samples. Statistical analysis was performed with Two-Way ANOVA.

Considering that the downregulation of nutrient-sensing pathways also contributes to CR-induced pro-longevity in *Sc*, we investigated the regulation of TOR and PKA pathway genes in MAT**a** and MATα cells. Our findings showed that, while TOR pathway genes remained unchanged in KN99α under CR (Fig. 3a), JEC21 exhibited minimal upregulation of certain genes such as *TOR1*, *AVO1*, *AVO2*, and *TSC2* (Fig. 3c). Conversely, both MAT**a** strains KN99**a** and JEC20 displayed mostly downregulation of TOR pathway genes (Fig. 3b and d) except *TAP42*, a downstream effector of the TOR complex that was upregulated in KN99**a** (Fig. 3b). The *Δsir2* mutants of both mating types exhibited no changes in the regulation of TOR pathway-associated genes (Fig. S3), suggesting that the TOR pathway does not compensate for the deletion of *SIR2* to increase the RLS. Transcriptional assessments of PKA pathway genes revealed mostly a lack of expression levels in both MATα and MAT**a** cells, except for the downregulation of *GPG1* in both mating types and *PKA2* in JEC21 and KN99**a** (Fig. 3e–h). These results demonstrate that the TOR pathway is downregulated only in MAT**a** strains during CR, possibly leading to the CR-mediated RLS extension in MAT**a** strains.

### Impact of calorie restriction on mitochondrial function

**Figure 4:**
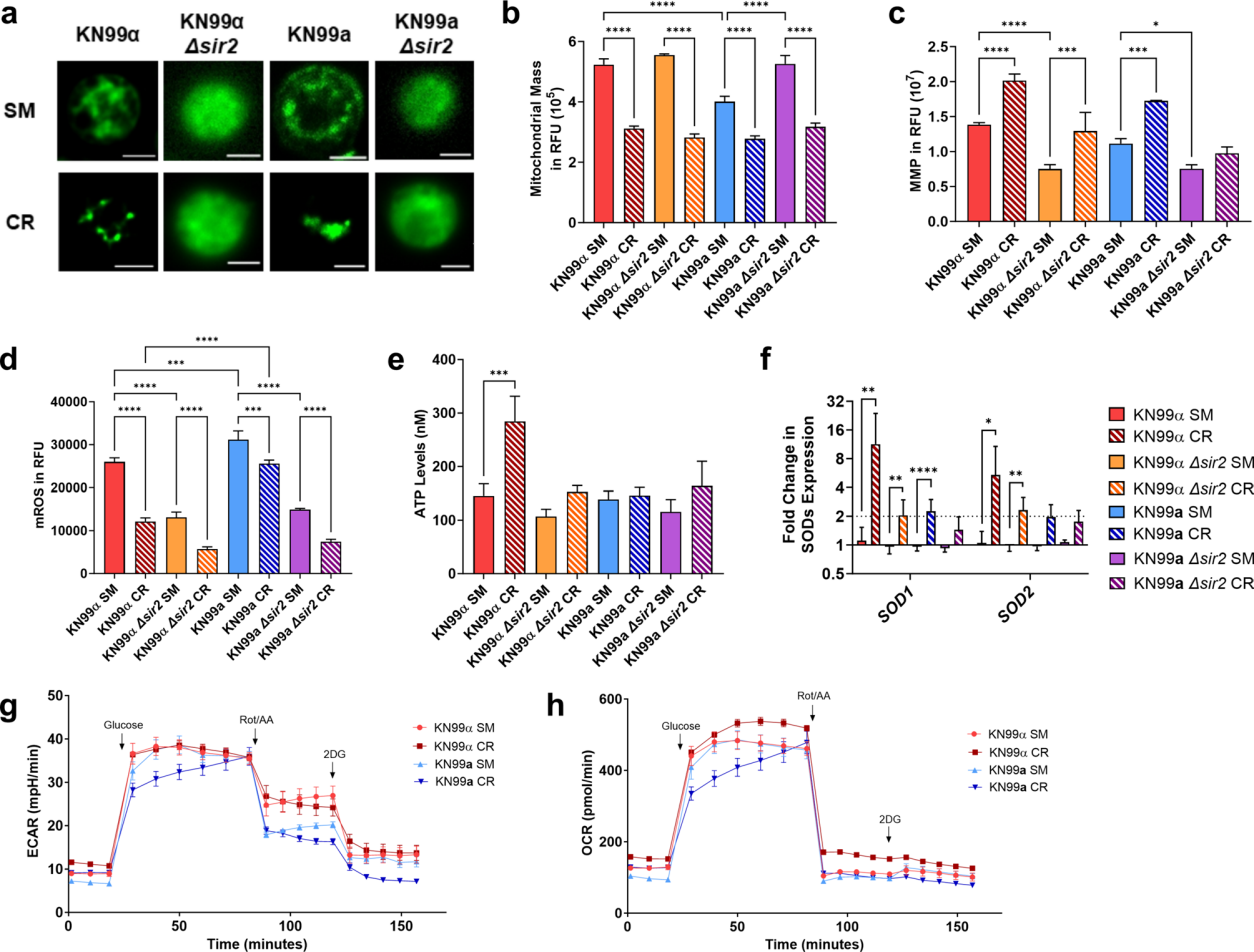
CR Affects Mitochondrial Function. (**a**) Cells stained with MitoTracker Green dye were observed in deconvoluted fluoresce studies. In SM, cells displayed tubular networks. In CR, mitochondria of KN99α (MATα) cells fragmented, and those of KN99**a** (MAT**a**) cells fragmented and clustered. *SIR2* deletion led to dispersed morphologies in both conditions. (**b**) KN99**a** exhibited lower mitochondrial mass than KN99α in SM media (*p* < 0.0001). CR lowered mitochondrial mass in all strains (*p* < 0.0001) as measured by MitoTracker Green dye. (**c**) Mitochondrial Membrane Potential (MMP) was comparable between KN99α and KN99**a** in SM. CR increased MMP in KN99**a** (*p* = 0.0001) and KN99α (*p* < 0.0001), as measured by JC-1 dye. *SIR2* deletion decreased MMP in both strains (*p* < 0.0001 and *p* = 0.0197). (**d**) Under CR, mitochondrial reactive oxygen species (mROS) levels were lower in KN99α (*p* < 0.0001) and KN99**a** (*p* = 0.0001) compared to SM, as measured with MitoSOX Red dye. Overall, mROS levels were higher in KN99**a** cells (SM *p* = 0.0003, CR *p* < 0.0001). *SIR2* deletion decreased mROS formation (*p* < 0.0001). (**e**) ATP levels were increased in KN99α under CR compared to SM (*p* = 0.0003), while ATP levels were unchanged in all other strains as measured by an ATP Bioluminescence assay Kit. (**f**) CR upregulated the cytoplasmic superoxide dismutase (*SOD1*) in KN99α (11.3-fold, *p* = 0.0063), KN99**a** (2.2-fold, *p* < 0.0001), and KN99α *Δsir2* (2.05-fold, *p* = 0.0075) compared to SM. The mitochondrial SOD (*SOD2*) was upregulated only in KN99α (5.39-fold, *p* = 0.027) and KN99α *Δsir2* (2.32-fold, *p* = 0.0012) during CR. qPCR was used to analyze transcription and *ACT1* was the internal control. The dotted lines signify a twofold up- or downregulation of the genes. (**g**,**h**) Extracellular acidification rate (ECAR) and oxygen consumption rate (OCR) profiles were generated by injection of glucose, Rotenone and Antimycin A (Rot/AA), and 2-deoxyglucose (2DG). KN99**a** has a lower basal metabolism and glycolysis than KN99α. Assays were performed in biological triplicate and error bars signify standard deviation between samples. Statistical analysis was performed with Student’s *t*-test with Welch’s correction and One-Way ANOVA.

In *Sc*, growth under CR is accompanied by mitochondrial biogenesis and increased respiration, resulting in decreased oxidative damage and a prolonged lifespan^30,31^. As the sirtuins have been associated with mitochondrial function and morphology^32,33^, we compared the mitochondrial function of both mating types exposed to SM and CR growth conditions. Changes in mitochondrial morphology are caused by dynamic reorganization of the mitochondrial network and play a pivotal role in responding to glucose starvation. We therefore investigated the mitochondrial morphology utilizing the mitochondrion-specific fluorescent dye MitoTracker Green FM, and deconvolution fluorescence microscopy. These data show distinct differences in mitochondrial morphologies between KN99α, KN99**a** cells, and their respective *Δsir2* mutants. Specifically, tubular mitochondrial networks were observed in both mating types grown in SM medium, while glucose starvation led to fragmentation, which was more pronounced with centralized clustering of mitochondria in KN99**a** when compared to KN99α. Interestingly, loss of *SIR2* was associated with diffuse mitochondrial morphology in both mating types grown in SM and CR conditions (Fig. 4a). Mitochondrial mass was measured by MitoTracker Green FM dye and decreased by 30–50% under CR exposure, whereby the KN99**a** strain had a lower mass in SM media (Fig. 4b).

Mitochondrial membrane potential (MMP) was measured by staining the cells with the JC-1 dye. MMP regulates respiration rates and correlates with mROS^34,35^. MMP was comparable between KN99α and KN99**a** cells, and loss of *SIR2* was associated with lower MMP. Under CR, MMP increased for all strains except the *Δ*sir2 mutant of KN99**a** (Fig. 4c). The respiratory process both generates ATP and inadvertently produces mitochondrial reactive oxygen species (mROS) which can be measured by the MitoSOX Red dye. Our data demonstrated higher mROS levels in KN99**a** compared to KN99α. Consistent with higher MMP, glucose limitation led to a reduction in mROS for both strains, whereas the lower MMP was associated with decreased mROS formation in the *Δsir2* mutants (Fig. 4d). It is noteworthy that only KN99α produced increased levels of ATP when grown under CR (145 nM to 284 nM), while no augmentation of ATP levels was seen in KN99**a** or the *Δsir2* mutant strains (Fig. 4e). These data suggest increased oxidative damage under CR stress in KN99**a** may impair ATP production. Corroborating this interpretation, CR-induced upregulation of both superoxide dismutases (SOD) was found in KN99α (*SOD1*: 11.3-fold, *SOD2*: 5.3-fold), whereas CR in KN99**a** resulted in minimal upregulation of cytoplasmic SOD (*SOD1*: 2.2-fold) and no differential regulation of mitochondrial SOD. In the KN99α *Δsir2* mutant, moderate upregulation of *SOD1* (twofold) and *SOD2* (2.3-fold) was observed during CR, while no differential regulation was observed in the KN99**a** *Δsir2* mutant (Fig. 4f).

Lastly, to gain further insight into the metabolism of the two mating types, we analyzed the extracellular acidification rate (ECAR) and oxygen consumption rate (OCR) as a measure of glycolysis and oxidative phosphorylation, respectively. KN99**a** exhibited lower basal metabolism in both ECAR and OCR measurements (Fig. 4g and h), indicating lower rates of glycolysis and respiration. The injection of glucose increased metabolism in both strains, with KN99**a** CR showing slower glucose metabolization. Inhibition of respiration by Rotenone and Antimycin A (Rot/AA) reduced respiration rates of both strains (Fig. 4h) and revealed that KN99**a** has lower glycolysis levels compared to KN99α (Fig. 4g). No difference in glycolysis rate was found between SM and CR growth conditions. We conclude from these data that under CR, mitochondrial function of MAT**a** cells is more compromised than that of MATα.

### Effect of age-dependent tolerance to FLC to MATα and MATa under CR

**Figure 5:**
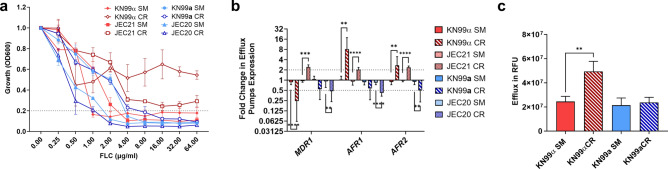
Efflux-mediated FLC Tolerance does not Occur in MAT**a** cells. (**a**) Fluconazole (FLC) minimum inhibitory concentration (MIC) assay demonstrated that KN99**a** and JEC20 cells (MAT**a**) were not tolerant to FLC during CR conditions compared to SM media, while KN99α (MATα) and JEC21 (MATα) showed tolerance (**b**) ABC transporter (*AFR1*, *AFR2*, and *MDR1*) expression was downregulated in JEC20 (*AFR1* 0.42-fold, *p* = 0.0058 and *MDR1* 0.47-fold, *p* = 0.0007) and KN99**a** (*AFR2* 0.49-fold, *p* = 0.0037) in CR conditions compared to SM media. KN99α upregulated *AFR1* (8.17-fold, *p* = 0.0038) and *AFR2* (2.7-fold, *p* = 0.002), and downregulated *MDR1* (0.24-fold, *p* < 0.0001). JEC21 upregulated all three transporters (*MDR1* 2.43-fold, *p* = 0.00014; *AFR1* 2.03-fold, *p* < 0.0001; *AFR2* 2.38-fold, *p* < 0.0001). qPCR was used to analyze expression and *ACT1* was used as an internal control. The dotted lines signify a twofold up- or downregulation of the genes. (**c**) Rhodamine 6G fluorescent dye efflux exhibited an increase in efflux in KN99α during CR (*p* = 0.0015) compared to SM media, while KN99**a** had no increase in efflux. Assays were performed in biological triplicate and error bars signify standard deviation between samples. Statistical analysis was performed with Student’s *t*-test with Welch’s correction and One-Way ANOVA.

Mitochondria provide the majority of cellular ATP, which fuels the energy-dependent ATP-binding cassette (ABC) transporters. Earlier studies with KN99α have indicated that with advanced generational age or when exposed to CR, *Cn* exhibits augmented tolerance to FLC mediated by enhanced efflux pump activity of the ABC transporters^22,36^. Given the differences in ATP production between mating types, we compared the FLC tolerance between the two mating types. In contrast to KN99α, and despite comparable baseline susceptibility, the FLC tolerance of the MAT**a** strains KN99**a** or JEC20 did not change when grown in CR conditions (Fig. 5a). Since there is a difference in ATP production when *SIR2* is deleted, we further analyzed FLC susceptibility in the KN99α *Δsir2* mutant. Deletion of *SIR2* in KN99α led to a higher susceptibility to FLC under CR, although 80% inhibition was not attained (Fig. S4).

As previously described^22^, ABC transporters were upregulated in KN99α (*AFR1*: 8.1-fold and *AFR2*: 2.7-fold) and JEC21 (*MDR1*: 2.4-fold, *AFR1*: twofold, and *AFR2*: 2.3-fold). We quantified their transcription in MAT**a** strains and found downregulation of *AFR2* (0.49-fold) in KN99**a** cells and *AFR1* (0.42-fold) and *MDR1* (0.47-fold) in JEC20 under CR (Fig. 5b). Consistent with this finding, a Rhodamine 6G fluorescent dye efflux assay showed increased efflux in KN99α in CR media, while no increase in efflux was observed in KN99**a** (Fig. 5c). These results suggest that decreased ATP production in MAT**a** cells results in loss of CR-induced upregulation of efflux pumps and FLC tolerance.

### Comprehensive transcriptome analysis in MATα and MATa cells under CR

**Figure 6:**
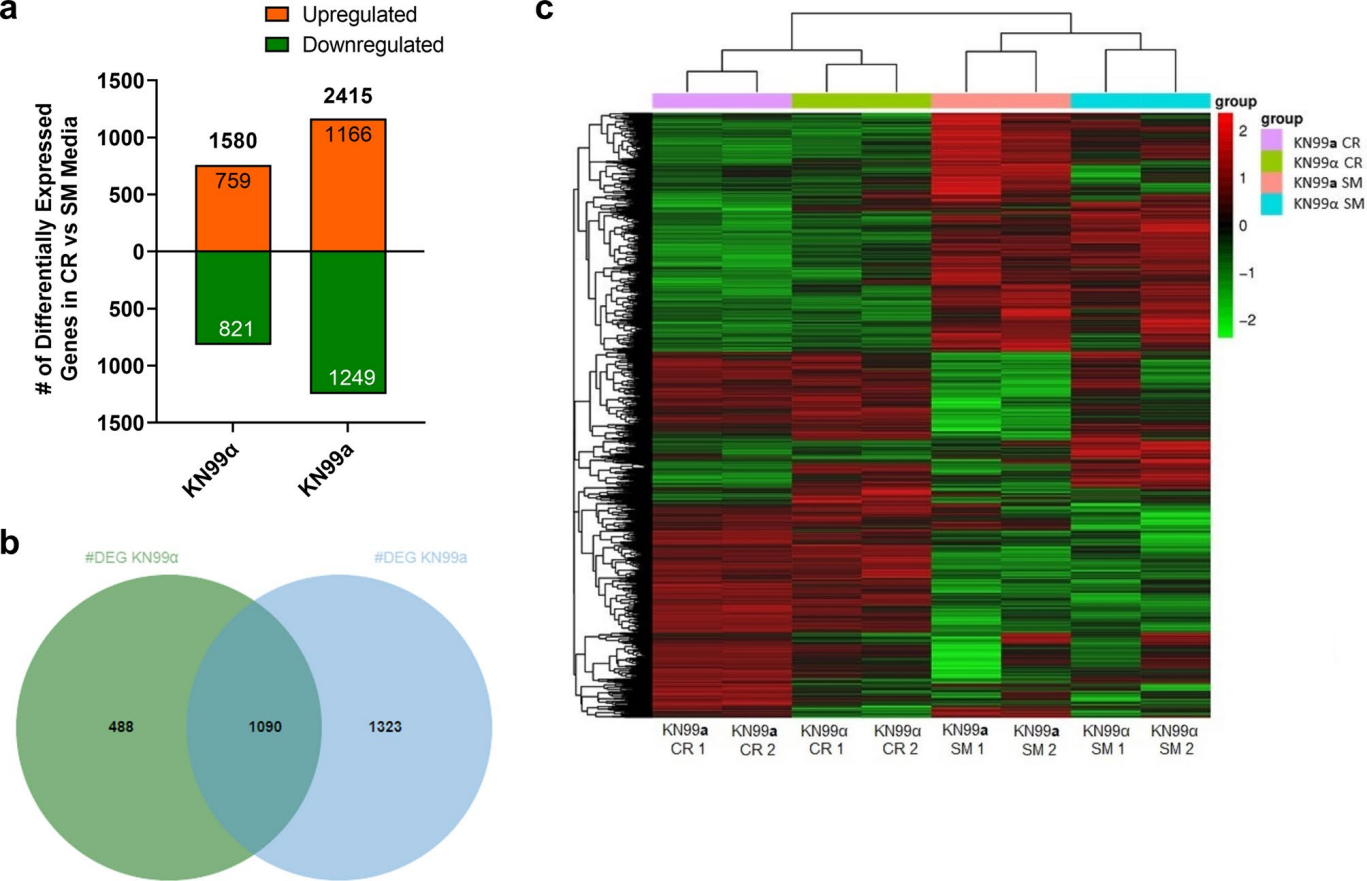
Transcriptomic Changes During Calorie Restriction are Different between KN99α and KN99**a**. RNAseq of KN99α (MATα) and KN99**a** (MAT**a**) cells was performed under SM and CR growth conditions. (a) KN99α under CR differentially expressed 1580 genes (759 upregulated and 821 downregulated), while KN99**a** differentially expressed 2415 genes (1166 upregulated and 1249 downregulated). (**b**) Venn diagram of differentially expressed genes (DEG) between KN99α and KN99**a** demonstrated that 1090 DEGs were common between the two strains, while 1323 were unique to KN99**a** and 488 were unique to KN99α. Image generated with jvenn software. (**c**) The heatmap showed that duplicates of the same sample (identified by 1 and 2 at the bottom axis) clustered together and that CR samples have a distinctly different expression profile than SM-grown cells.

Lastly, an RNAseq of KN99α and KN99**a** cells grown under SM and CR was performed to uncover additional differences in CR-induced regulation. Consistent with attenuated growth, CR led to more downregulation of gene transcription in both mating types. KN99α cells altered transcription of 1,580 genes under CR, while KN99**a** altered transcription of 2415 genes (Fig. 6a). Although both mating types shared 1090 genes, many differentially expressed genes (DEGs) were mating type-specific (Fig. 6b). Heatmap analysis confirmed correlation and clusters in duplicates (Fig. 6c). Gene ontology (GO) classification and enrichment analysis showed different responses to CR. In KN99α, CR led to an increase in 55 molecular functions and 45 biological processes, and a decrease in 27 functions and 52 processes whereas under CR, KN99**a** increased 46 functions and 39 processes but decreased 65 functions and 90 processes (summarized in Fig. S5).

While overlapping GO categories such as transport, calcium homeostasis, and phosphorylation were noted, the majority of enriched categories were either specific to KN99α or KN99**a**. For KN99α, upregulated GO categories included NAD/NAD+ binding, which correlates with the activity of NAD+-dependent sirtuins. Furthermore, antioxidant activity, ROS metabolic processes, as well as superoxide dismutases, catalases, and cytochrome c peroxidases that mediate the breakdown of H_2_O_2_ were upregulated (Fig. S5). In KN99**a**, categories related to lipid metabolism, such as fatty acid synthase activity, cellular lipid metabolism, sphingolipid metabolism, and lipid glycosylation, were significantly enriched (Fig. S5). Although the genes of catalase and cytochrome c peroxidase were upregulated, the antioxidant activity GO category was downregulated in KN99**a**, which included sulfiredoxin, cytochrome c peroxidase, catalase, peroxiredoxin, thioredoxin reductase, and thiol-specific antioxidant protein 3 genes (Fig. S5).

Importantly, CR induced altered transcription of many mitochondrial genes in both mating types. In KN99α, 43 mitochondrial genes related to the electron transport chain and ATP synthesis were upregulated. These included 17 genes encoding for subunits of Complex I (NADH dehydrogenase), 4 for Complex II (succinate dehydrogenase), 3 for Complex III (cytochrome *bc*), one for Complex IV (cytochrome c oxidase) and Complex V (ATP synthase) that were upregulated. Most of the 10 mitochondrial genes downregulated in KN99α by CR were associated with mitochondrial transport. In KN99**a**, 34 upregulated mitochondrial genes included 8 encoding for genes of Complex I, 3 for Complex II, and 2 for both Complex III and Complex IV subunits. Interestingly, 2 genes encoding Complex I and one for Complex IV subunits were downregulated, along with 5 genes encoding for the ATP synthase of Complex V. CR also increased the transcription of 13 mitochondrial transporter genes, while decreasing the transcription of 8 transporters and 3 genes related to mitochondrial morphology in KN99**a**. Together, the present findings provide further evidence that MATα and MAT**a** cells respond differently to CR stress. The mating types show distinctly different transcriptomes, including differences in the regulation of mitochondrial function and, specifically, ATP synthesis.

## Discussion

The present study addressed the divergent responses of *Cn* mating types to low glucose stress conditions. We found that *Cn* MATα cells upregulated *SIR2* and other sirtuins, while MAT**a** cells downregulated the TOR pathway, suggesting that the pro-longevity effect in the setting of CR is differently regulated between mating types. This difference in regulation impacts mitochondrial function and drug resistance in a mating type-dependent manner. KN99**a** cells exhibit higher oxidative damage with increased mROS resulting in compromised mitochondria function and decreased ATP production. As a result, MAT**a** does not exhibit enhanced FLC tolerance in a glucose-limiting environment. These results suggest that MAT**a** cells display inferior stress and pro-longevity response to CR, possibly explaining why this mating type is less prevalent.

While lifespan extension is a common outcome of CR, it is not universal. In fact, both shortened and unchanged RLS have been observed in *Cn* and other yeast species^37^. In *Sc*, studies using strains PSY316 and BY4742 have demonstrated RLS extension under CR^28,38–40^, while investigations involving the W303R strain have yielded different results^41^. Previous studies have also documented variable RLS responses in clinical *Cn* MATα strains when subjected to CR^37^. Notably, the pro-longevity effect of CR in most of the investigated *Cn* strains appears more pronounced compared to *Sc*, with RLS extension reaching up to 400%. This substantial difference may be attributed to differences in glucose metabolism of the two yeasts. Unlike Crabtree-positive *Sc*, which switches from fermentation to respiration in the presence of oxygen and low glucose concentrations, Crabtree-negative *Cn* primarily relies on respiration, requiring finer control over their metabolic responses^38,42^.

Sir2 and its homologs have an established role in modulating lifespan in *Sc* and *Cn*^23,43^. In contrast to *Sc*, where Sir2 regulates chromatin silencing at the mating locus, telomeres, and rDNA repeats, in *Cn*, Sir2 predominantly associates with tRNA genes and the rDNA region, with no interaction with the *MAT* locus or telomeric regions^44–47^. Our findings highlight differences in Sir2 regulation between mating types of *Cn* exposed to CR. While transcription of *SIR2* and other sirtuins was upregulated in KN99α under CR, it did not affect their transcription in KN99**a.** Additionally, CR-induced RLS prolongation was *SIR2*-dependent only in KN99α, and not in KN99**a**, where *Δsir2* continued to exhibit a prolonged RLS under CR. This result confirmed previous findings that H99 RLS extension is *SIR2*-dependent under low glucose conditions^27^. These findings may indicate that, unlike KN99α, *SIR2* activity is not necessary for longevity in KN99**a**, playing only a secondary role. Similar findings have been described in *Sc* strains where the pro-longevity effect of CR is only *SIR2*-dependent in W303R but not in PSY316^23,48^. This data confirms that not all strains are *SIR2*-dependent for RLS extension, although no correlation with mating types was described in *Sc*.

The role of other sirtuins was entertained. For *Cn* *Δsir2, Δhst3,* and *Δhst4* phenotypical changes and hypovirulence have been reported^47^. Unlike *SIR2*, the deletion of *HST3* and *HST4* did not affect RLS, indicating that they alone do not play a role in longevity. Since the sirtuins are gene silencers, it has been proposed in *Cn* that *SIR2* deletion leads to loss of heritable epigenetic information^47^, explaining the reported failure to successfully complement their knockout mutants to restore wild-type activity. To account for the lack of complemented strains, we generated two independent *Δsir2* mutants for KN99α and KN99**a**, which demonstrated that the phenotype was consistent.

Decreased TOR activity in *Sc*, by null mutants or during induction by CR, prolongs their lifespans^28,49^. Consistent with lack of *SIR2* regulation we found the TOR pathway was downregulated in MAT**a** strains, but mostly unchanged in MATα strains. This suggests diverse roles of the TOR pathway in the longevity regulation of MAT**a** and MATα strains. Our data supports the concept that *SIR2* regulation is not the only response option to CR. While MATα cells are dependent on the function of *SIR2* for RLS extension, MAT**a** cells may be dependent on the TOR pathway downregulation. Similar to *SIR2*, the pro-longevity effect of decreased TOR activity appears independent of mating type in *Sc*^28,48^. Unfortunately, *Cn* cannot be studied through a genetic approach because the singular *TOR1* gene of *Cn* is essential, unlike *Sc* which has two TOR genes^50^.

Mitochondrial dysfunction is a hallmark of aging, as damaged mitochondria are asymmetrically segregated to mother cells, which is associated with higher ROS levels and a lower redox potential^51^. Increased mitochondrial biogenesis and respiration, as well as decreased oxidative stress, are achieved through CR in *Sc*^30,31^. In contrast to other yeasts, glycolysis and respiration are highly interdependent in Crabtree-negative *Cn*^52^. As a result of its metabolic inflexibility, *Cn* needs to fine-tune respiration and cannot live without mitochondrial function^38,42^. Under glucose restriction, KN99**a** exhibited lower metabolic activity than KN99α, especially when glycolysis was analyzed with Seahorse. Since *Cn* cannot regenerate NAD+ through fermentation, the cells are unable to meet their ATP demands, resulting in stalled glycolysis^52^. Usually, NAD+ is reduced into NADH during the glycolytic metabolic process, which will generate ATP^53^. Lower glycolysis levels may be the result of a smaller NAD+ pool, which would generate less ATP in KN99**a** cells. Increased NAD+ binding GO term in KN99α could also indicate higher NAD+ availability. Depletion of cytoplasmic NAD+ can block glycolysis^54^ and cause mitochondrial dysfunction, with a decline in energy production and ROS accumulation, producing high oxidative stress^55^.

In the mitochondria of *Cn,* oxygen is essential for oxidative phosphorylation and ATP production. Furthermore, mitochondrial impairment can lead to oxygen not receiving a full complement of electrons for reduction, ultimately resulting in harmful ROS accumulation^56,57^. Higher MMP levels in *Cn* under CR indicate sufficient mitochondrial health to facilitate enhanced production of ATP^58^. However, CR-driven increase in ATP production was only observed in KN99α and not in KN99**a** cells. The RNAseq results explain these findings. During CR, subunits of all electron transport chain complexes were found to be upregulated in KN99α. Despite the upregulation of genes that encode subunits of complexes I-IV, KN99**a** did not enrich any GO terms related to mitochondrial respiration. This is likely the consequence of the downregulation of genes encoding for subunits of complex V, which is responsible for ATP synthesis in mitochondria. mROS levels were also consistently higher in KN99**a** compared to KN99α, indicating marked differences in mitochondrial function between mating types. The SODs catalyze the breakdown of ROS into hydrogen peroxide, which is then converted into oxygen and water^59^. Higher mROS levels in KN99**a** were consistent with lower transcription of mitochondrial and cytoplasmic SOD compared to KN99α. Lower mROS levels in KN99α under CR can be attributed to the upregulation of two SODs, two cytochrome c peroxidases, and a catalase. KN99**a** transcriptome indicated enhanced transcription of hydrogen peroxide metabolism-associated genes, and downregulation of antioxidant activity, suggesting a compromised antioxidant response.

During permissive, non-stressful growth, the *Cn* mitochondria are present primarily in a reticular morphology^60,61^. Upon encountering mitochondrial stress, such as CR, mitochondria undergo fusion and fission, enriching tubular and fragmented morphologies^61^. Mitochondrial fusion permits sharing of mitochondrial DNA, and mitochondrial fission facilitates removal of damaged mitochondria. The mitochondria of KN99α underwent fragmentation to counteract the stress of CR, but the mitochondria of KN99**a** displayed fragmentation with a clustered morphology consistent with more pronounced stress. Taken together, these phenotypes demonstrate that MAT**a** cells display higher oxidative stress and mitochondrial damage than their MATα counterparts. As mitochondria are essential for *Cn* survival, these differences in stress response may influence mating type prevalence.

Deletion of *SIR2* influenced mitochondrial function and morphology in both mating types. The *Δsir2* mutants displayed an extremely diffuse phenotype, with loss of the tubular and fragmented phenotypes observed in the wild-type strains. This is consistent with the association of mammalian sirtuins with the modulation of fusion/fission of the mitochondrial network. Inhibition of SIRT1 and SIRT3 alters the mitochondrial morphology by preventing fusion/fission^33,62^. Morphology regulation is achieved by modulation of the fission dynamin-1-like protein (Drp1) and actin cytoskeleton^33,63–65^. *SIR2* deletion also led to decreased MMP, ROS, and ATP production. Mammalian sirtuins enhance oxidative phosphorylation, electron transport chain, and ATP formation^32,66,67^, which is consistent with the diminished mitochondrial function observed in *Δsir2* mutants of *Cn*.

Mitochondrial ATP hydrolysis is the energy source for the ABC transporters superfamily. Mitochondrial respiratory activity is directly associated with efflux pump-mediated resistance in several fungi^22,68^. Generally, an increase in respiration leads to a higher ATP generation and transport to the cytoplasm, ensuring that drug efflux pumps gain more available ATP to increase efflux^68^. Compromised ATP production resulted in reduced efflux pump activity in KN99**a,** explaining why CR did not induce higher FLC tolerance in both MAT**a** strains, in contrast to MATα *Cn* strains. These results further indicate that MAT**a** strains respond to stress less effectively than MATα, as they can’t counteract the effect of FLC.

Congenic MAT**a**/MATα mating pairs are genetically identical except for their mating locus; therefore, this locus could explain the distinct responses to CR between mating types. The *MAT* locus harbors three transcription factors associated with carbon and nutrient metabolism, *ZNF1*α/**a**, *STE12*α/**a**, and *SXI1*α/2**a**. In *Cn*, published RNAseq data found *STE12*α upregulated in H99 (MATα) under CR (fold-change = 10.6)^27^, which we confirmed here in KN99α, but not in KN99**a**. In *Cn,* Ssn8 is the downstream target of *STE12*α and, in response to nutritional stress, regulates 75 genes involved in nutrient scavenging^16^. Deletion of *STE12*α in *Cn* resulted in decreased activity of SOD enzymes, suggesting a link between the *MAT* locus and SOD regulation^69^, which could explain the difference in ROS levels between mating types. Differential regulation of these transcription factors or their downstream targets may contribute to several differences between mating types.

In summary, CR exerts a diverse influence on the longevity regulation of *Cn* mating types, with MATα cells being *SIR2*-dependent and MAT**a** cells being *SIR2*-independent, possibly relying on the downregulation of the TOR pathway. This difference in CR response affects mitochondrial function, morphology, and drug resistance in a mating type-dependent manner. MAT**a** strains exhibit higher levels of oxidative stress and mitochondrial damage, as well as a decreased ability to combat antifungal drugs under CR stress. It is possible that better stress response mechanisms could be responsible for the higher prevalence and greater virulence of MATα cells. Our data further encourage efforts to characterize longevity and stress responses in *Cn* cells.

## Materials and methods

### Strains and media

*Cn* strains KN99α (MATα, serotype A), KN99**a** (MAT**a**, serotype A), JEC21 (MATα, serotype D), JEC20 (MAT**a**, serotype D), KN99α *Δsir2*-1, KN99α *Δsir2*-2, KN99**a** *Δsir2*-1, KN99**a** *Δsir2*-2, H99 (MATα, serotype A), *Δhst2, Δhst3, Δhst4,* and *Δhst5* were cultured in synthetic media (SM, 1.7 g yeast nitrogen base without amino acids, 1 g drop out mix, 4 mL ethanol, 5 g (NH_4_)_2_SO_4_, 3.3 g NaCl) with either 2% glucose (SM) or 0.05% glucose (calorie restriction media, CR). The KN99α *Δsir2*-1 strain was derived from the Madhani knockout collection, which is managed by the Fungal Genetics Stock Center. All strains used in this study (Table S1) were maintained as 30% glycerol stocks and stored at − 80 °C for future use.

### Deletion of SIR2 in KN99a and KN99α

*SIR2* deletion was performed using the TRACE CRISPR-Cas9 system as previously described^70–72^. The Cas9 cassette was amplified from the codon-optimized pMH21 plasmid (pRS316-P_TEF1_-CAS9_optimized; oligonucleotides M13 F and M13 R) with an Ex Taq DNA-Polymerase. To make the sgRNA construct, the *Cryptococcus* native U6 promoter was amplified from the BHM2329 plasmid (pRS316-promoterCnU6-sgRNAscaffold-6T; oligonucleotides C8573 and sgRNA R). The reverse oligo contains a 20 bp reverse complement sequence of the genomic target for the Cas9 enzyme (gRNA). The gRNA (upstream of PAM and excludes PAM from the oligonucleotide) was designed using the Eukaryotic Pathogen CRISPR guide RNA/DNA Design Tool (http://grna.ctegd.uga.edu/) by searching for a sequence within 150 bp of the 5ʹ UTR region up to the start codon of *SIR2*. The scaffold/terminator sequence was also amplified from the BHM2329 plasmid (oligonucleotides sgRNA F and C8574) with a forward oligo that contains the forward sequence of the gRNA. All fragments were run in a 0.8% agarose gel and gel purified using the QIAquick Gel Extraction Kit. Equal volumes of the U6 and scaffold/terminator fragments were mixed and used as a template for a fusion PCR using Ex Taq Polymerase (oligonucleotides C8573 and C8574). The resulting fragment was gel extracted and purified. The DNA construct to be integrated was designed to contain 50 bp of homology to the 5ʹ UTR of *SIR2* + HYG^R^ (hygromycin resistance gene) + 50 bp of homology to the 3ʹ UTR of *SIR2*. This was achieved by amplifying the HYG^R^ gene from the pPZP-HYG plasmid with oligonucleotides containing the homology arms to *SIR2*. The fragment was gel extracted and purified. Transformation by electroporation was performed by growing KN99**a** and KN99α overnight and inoculating them into 100 mL YPD (OD_600_ = 0.2). They were grown for 4–5 h (OD_600_ = 0.6–1.0), centrifuged, and washed twice in ice-cold water. The cells were then resuspended in 10 mL of ice-cold electroporation buffer (EB; 10 mM Tris–HCl pH 7.5, 1 mM MgCl_2_, 270 mM sucrose) with 1 mM DTT and incubated on ice for 1 h. Cells were centrifuged and resuspended in 220–250 μL of EB with 1 mM DTT. PCR products were mixed with the cells as follows: 50 μL of cells, 2 μg of Cas9 cassette, 700 ng of sgRNA cassette, and 4 μg of deletion cassette. They were transferred to a pre-cooled 2 mm gap electroporation cuvette, and a negative control with only cells was also transferred into a separate cuvette. Cells were transformed in a MicroPulser Electroporator at the following settings: 2 kv, 4 ms. Following transformation, cells were recovered in 1 mL of YPD media and incubated at 30 °C for 1–2 h. Cells were plated in HYG YPD agar and incubated for 2–3 days. Positive colonies were passaged in selective media three times. For confirmation, DNA was extracted, and PCR was performed to confirm gene deletion (oligonucleotides SIR2 F and SIR R). Additionally, expression was determined by qPCR (oligonucleotides qPCR SIR2 F and qPCR SIR2 R). Oligonucleotides are described in Table S2.

### Replicative lifespan analysis

RLS was determined as previously established^73^. Briefly, cells were grown on SM or CR agar plates overnight. Individual 20–30 naïve cells were dissected under a tetrad dissection Axioscope A1 microscope. The RLS is defined as the number of times a mother cell buds before undergoing senescence, which is determined as 24 h without a budding event.

### Antifungal susceptibility

The minimum inhibitory concentration (MIC) was determined as previously described^74^. Briefly, 10^5^ cells/well of *Cn* strains were incubated with serially diluted fluconazole (FLC) for 4 days at 37 °C. The MIC was defined as the concentration that inhibits 80% of cell growth (MIC_80_) at OD_600_.

### Transcriptome analysis

Cells were grown overnight in SM or CR media. RNA extraction, cDNA conversion, and qPCR were performed as previously described^36^, following the manufacturers' guidelines. The oligonucleotides employed to analyze the gene expression of the sirtuins, TOR pathway, PKA pathway, SOD, and efflux pumps genes are described in Table S2. The housekeeping gene *ACT1* was used as an internal control. Data were calculated by the 2^−ΔΔCT^ method, as previously described^75^. Expression was considered significant above or below a twofold change threshold. The assay was conducted with 3–5 biological replicates.

### RNAseq analysis

KN99α and KN99**a** were grown overnight in SM or CR media. RNA was extracted using the RNAeasy Plus kit, following the manufacturer’s guidelines. RNASeq was performed by Novogene Co. Messenger RNA was purified from total RNA using poly-T oligo-attached magnetic beads and converted to cDNA. Libraries were clustered and sequenced on an Illumina NovaSeq 6000 platform, which utilizes a paired-end 150 bp sequencing strategy (PE150) according to the manufacturer's instructions. Raw data were processed by removing reads containing adapters, reads containing unknown nucleotides, and low-quality reads. Clean reads were aligned to the reference genome using Hisat2 v2.0.5. Differential expression analysis was performed employing the DESeq2 R package (1.20.0). Genes with a *p-*value <  = 0.05 and |log2FoldChange|> = 0.5 were assigned as differentially expressed. Gene ontology (GO) Enrichment analysis was performed by running differentially expressed genes through FungiDB, selecting for Biological Process (BP) and Molecular Function (MF) with a *p*-value cutoff of 0.05. Venn diagrams were generated using the jvenn software (http://jvenn.toulouse.inra.fr/app/index.html). Heatmap was generated by Novogene Co.

### Cellular ATP levels analysis

Measurement of cellular ATP levels was performed using ATP Bioluminescence Assay Kit following the manufacturer’s guidelines. Briefly, cells were cultured overnight in SM and CR media, and 10^7^ cells were employed in this assay. Cellular ATP was isolated using an alkaline lysis buffer and 5% trichloroacetic acid. They were vortexed for 15 min in the presence of sterile acid-washed glass beads to initiate lysis. The cell lysates were boiled for 10 min at 100 °C. A standard curve was produced using an ATP Standard, following the manufacturer’s guidelines. The samples and standard curve were mixed with a reaction mixture containing Assay Buffer, Substrate, and ATP Enzyme. The luminescence was immediately read on a plate reader. This assay was performed in triplicate.

### Measurement of mitochondrial activity and morphology

Mitochondrial reactive oxygen species (mROS) was measured by growing cells overnight in SM and CR media and staining 10^7^ cells with MitoSOX Red following the manufacturer’s guidelines as previously described, with modifications^22^. Cells were incubated with a final MitoSOX concentration of 5 μM. Mitochondrial membrane potential (MMP) was measured by growing cells overnight in SM and CR media and staining 10^7^ cells with JC-1 following the manufacturer’s protocol. JC-1 was resuspended in 383 μL of DMSO, at a concentration of 20 mM. The cells were resuspended in a final JC-1 concentration of 200 μM and incubated at 37 °C in the dark for 30 min. After staining, the cells were washed 3× with PBS, and 200 μL were loaded into a black clear-bottom 96-well plate. Fluorescence was measured at λ_ex_ of 535 nm and λ_em_ of 590 nm. Unstained cells were used as a negative control. Mitochondrial mass was measured by growing cells overnight in SM and CR media and staining 10^6^ cells with MitoTracker Green FM following the manufacturer’s protocol as previously described^22^. All assays were performed in biological triplicate. MitoTracker Green was further used to analyze mitochondrial morphology as previously described^22^.

### Rhodamine 6G efflux assay

KN99α and KN99**a** were grown overnight in SM and SM CR media and Rhodamine 6G assay was performed as previously reported^36^ using 2 × 10^7^ cells in a biological triplicate. Cells were incubated with 10 μM Rhodamine 6G for 30 min at 37 °C. Efflux was measured after 30 min.

### Seahorse analysis

A Glycolytic Rate assay was performed on a Seahorse Biosciences XFe96 extracellular flux analyzer for oxygen consumption rate (OCR) and extracellular acidification rate (ECAR) as previously described^52^. Briefly, cartridges were hydrated overnight in Agilent Seahorse XF calibrant. Cells were grown overnight in SM and CR media, and 180 μL of a 2 × 10^6^ cells/mL culture was loaded into each well. Assay solutions were injected to a final concentration of 20 mM glucose, 3.5 μM Rotenone/Antimycin A (Rot/AA), and 100 mM 2-deoxyglucose (2DG). Cells and solutions were prepared on Seahorse XF Base Medium supplemented with 2 mM l-glutamine and 5 mM HEPES pH 7.4. Each condition was measured with 8 replicates.

### Statistical analysis

Statistical analyses were performed using GraphPad Prism 9.0. The specific analyses are described in the figure legends.

### Supplementary Information

Supplementary Information.

## Data Availability

All data required to evaluate and understand the article are included. If additional data is required, the data are available on request from the correspondence author. RNAseq data is deposited in NCBI Gene Expression Omnibus (GEO) and can be accessed on the accession number GSE269512.
