# Supplementary material for: Distinct effect of calorie restriction between congenic mating types of Cryptococcus neoformans
Source: Sci Rep. 2024 Aug 6;14:18187. doi: 10.1038/s41598-024-69087-y (PMC11303771; doi:10.1038/s41598-024-69087-y)
Supplement: Supplementary file 1 — Supplementary Information. [file 41598_2024_69087_MOESM1_ESM.docx]

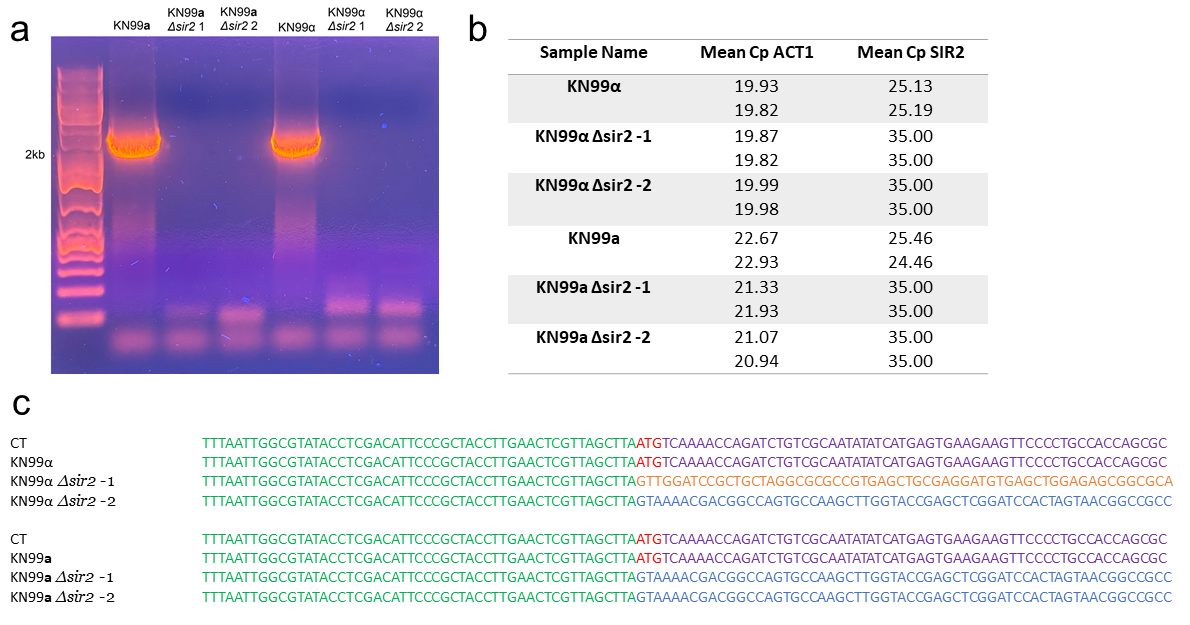


**Figure S1. Confirmation of *SIR2* Deletion in KN99α and KN99a.** (**a**) PCR showing amplification of the whole SIR2 gene in KN99α and KN99**a** wild-type controls and lack of *SIR2* amplification in the mutant strains. (**b**) qPCR analysis of mutant strains showed no amplification of *SIR2* in the *Δsir2* strains (Cp 35.00 is the assay detection limit), while parental strains amplified *SIR2* and *ACT1* control. (**c**) Sequencing of the insertion site of the deletion cassette in mutant strains, compared to wild-type sequencing (5’ UTR = green; ATG = red; SIR2 gene = purple; NAT resistance cassette = orange; HYG resistance cassette = blue).


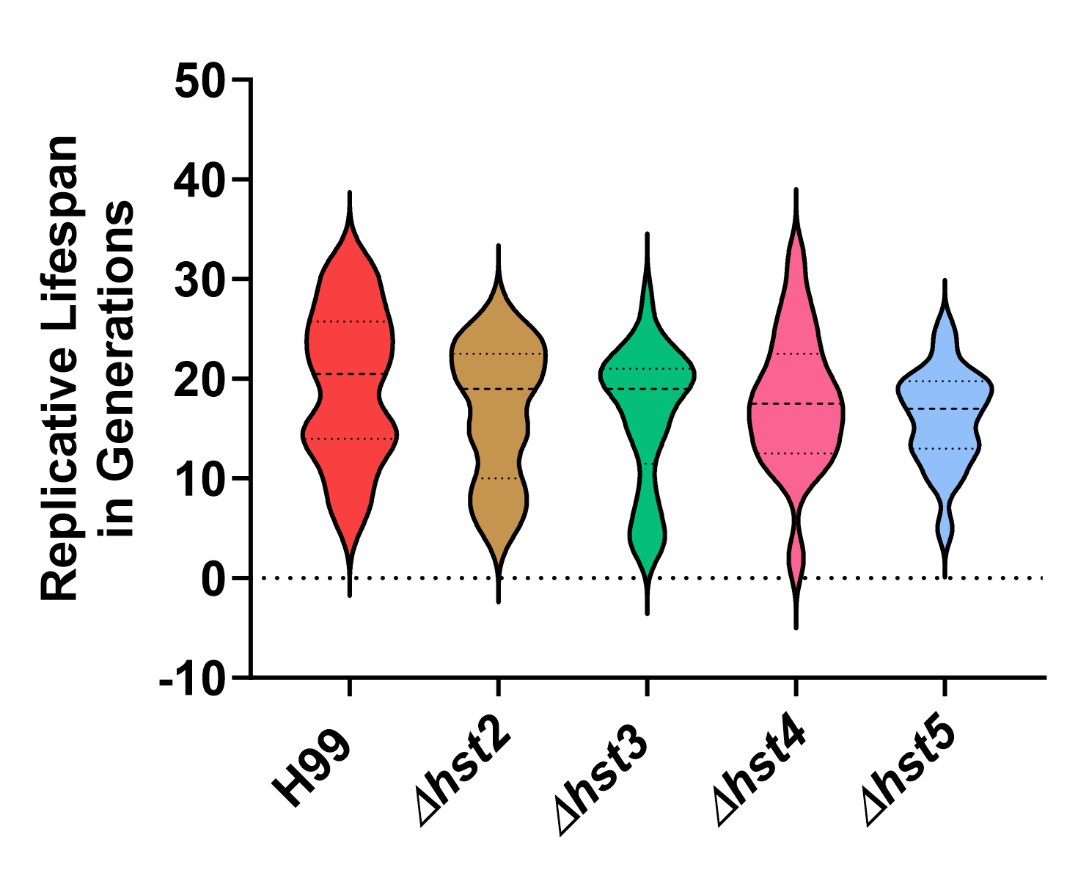


**Figure S2. RLS of Sirtuins Mutant Strains.** *Δhst2*, *Δhst3*, *Δhst4*, and *Δhst5* mutant strains showed no difference in RLS compared to wild-type control (MATα) in SM media.


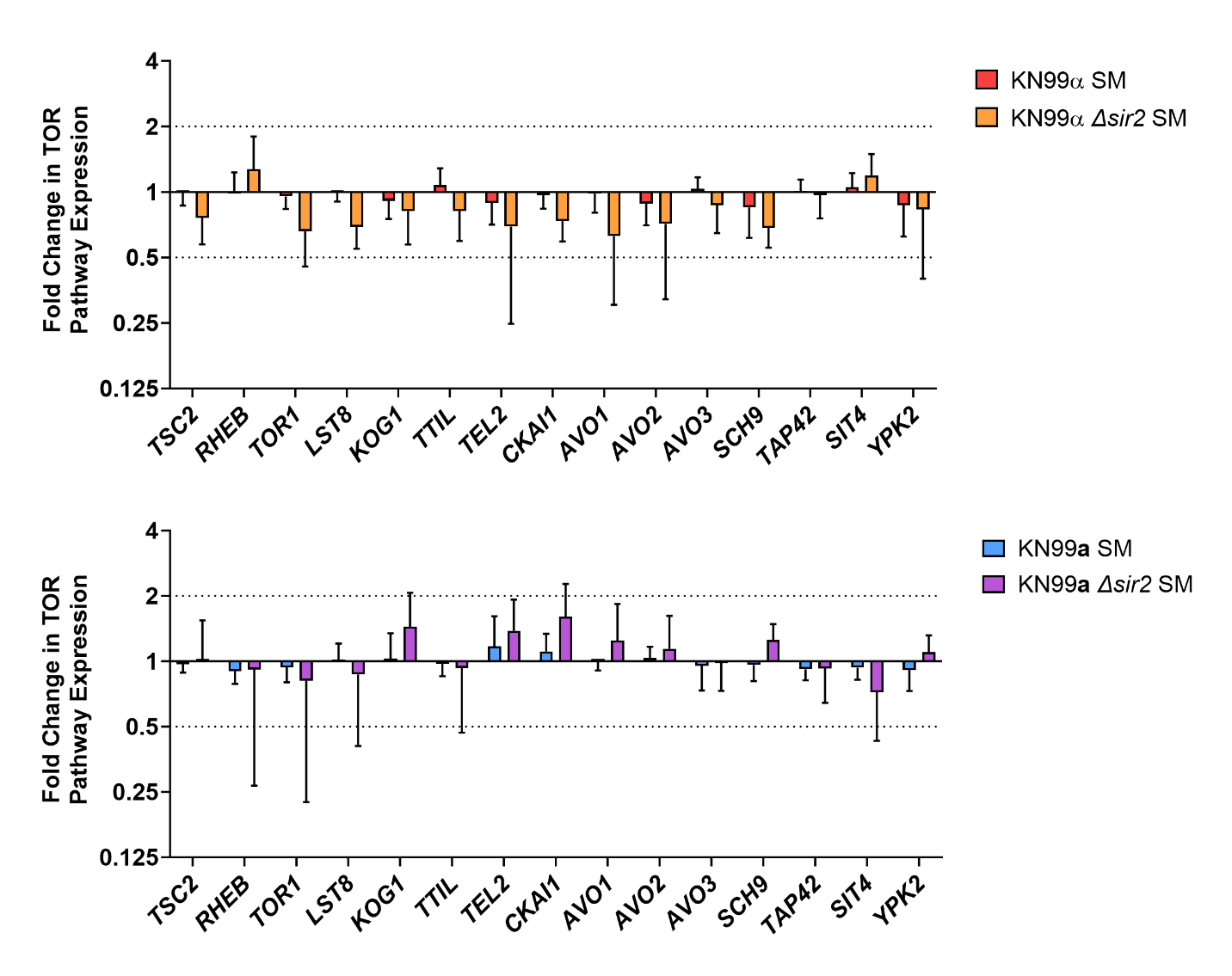


**Figure S3. TOR Pathway-associated Gene Expression in *Δsir2* Strains.** *Δsir2* strains on both KN99α and KN99**a** backgrounds showed no differential expression of the TOR pathway genes compared to their parental strains.


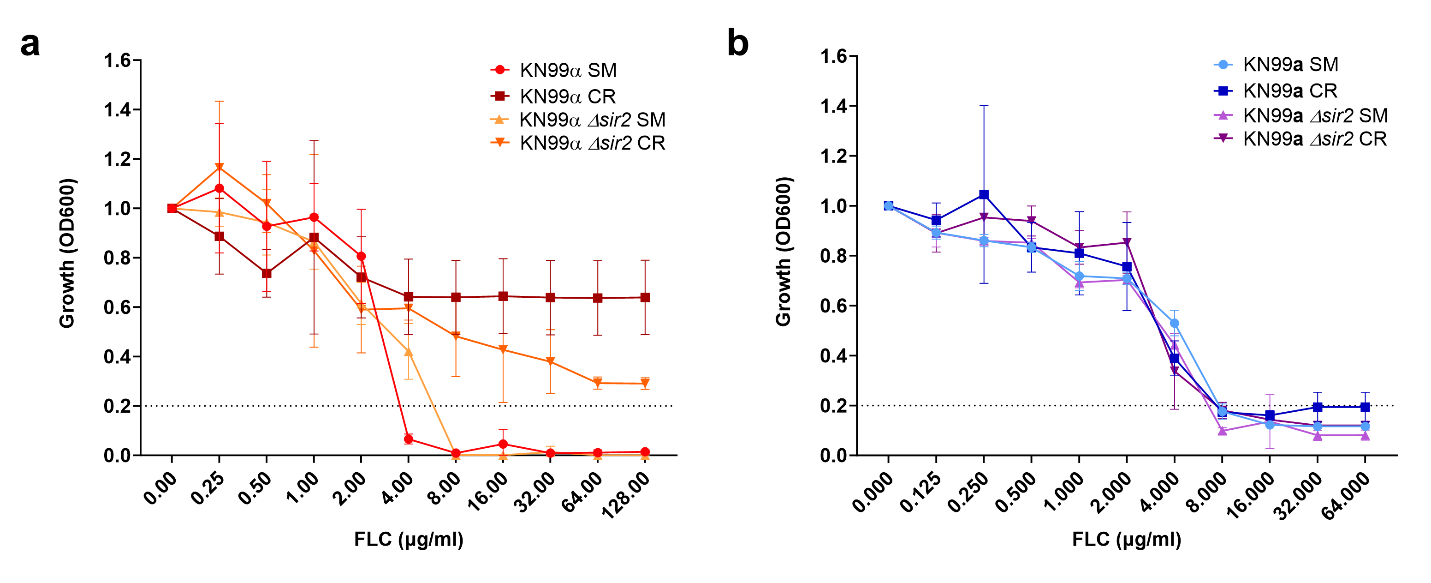


**Figure S4. Fluconazole Susceptibility in *Δsir2* Strains.** (**a**) KN99α *Δsir2* strain showed a slightly increased FLC susceptibility during CR compared to the wild-type MIC, although results remained above the 80% inhibition threshold. (**b**) KN99**a** *Δsir2* strain showed no difference from the parental strain.

**
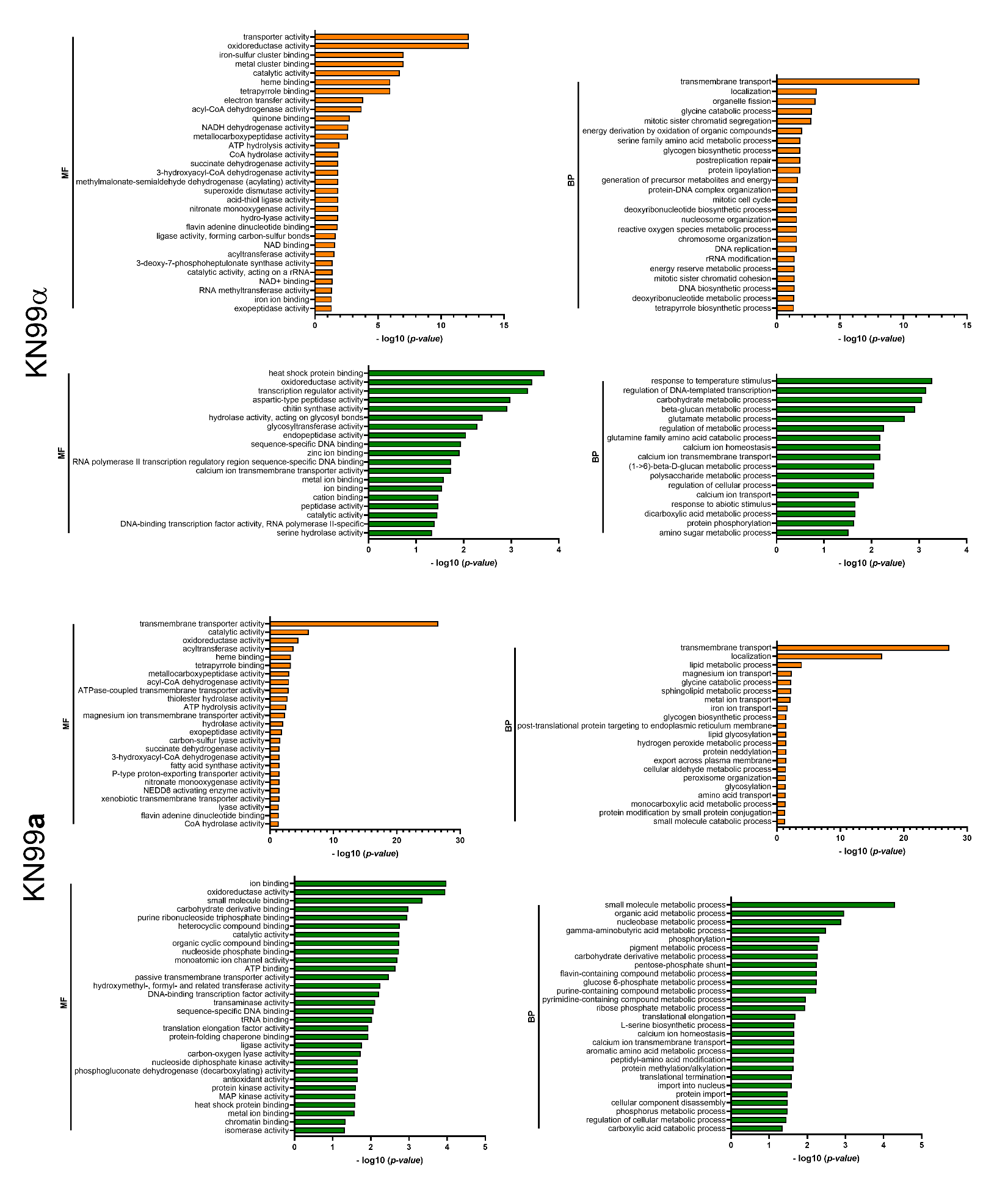
**

**Figure S5. Gene Ontology Analysis of DEGs.** Summary of upregulated (orange bars) and downregulated (green bars) biological processes (BP) and molecular functions (MF) of KN99α and KN99**a** during CR.

Table S1. Strains Table

| *C. neoformans* Strain | Serotype | Mating Type | Source or Reference |
| --- | --- | --- | --- |
| KN99α | A | MATα | WT |
| KN99a | A | MAT**a** | WT |
| JEC21 | D | MATα | WT |
| JEC20 | D | MAT**a** | WT |
| KN99α *Δsir2* -1 | A | MATα | Madhani knockout collection (Fungal Genetics Stock Center) |
| KN99α *Δsir2* -2 | A | MATα | This study |
| KN99a *Δsir2* -1 | A | MAT**a** | This study |
| KN99a *Δsir2* -2 | A | MAT**a** | This study |
| H99 | A | MATα | Arras, *et al*., 2017 |
| *Δhst2* | A | MATα | Arras, *et al*., 2017 |
| *Δhst3* | A | MATα | Arras, *et al*., 2017 |
| *Δhst4* | A | MATα | Arras, *et al*., 2017 |
| *Δhst5* | A | MATα | Arras, *et al*., 2017 |

Table S2. Oligonucleotides Table

| Primer Name | Sequence |
| --- | --- |
| M13 F | TGTAAAACGACGGCCAGTG |
| M13 R | GCGGATAACAATTTCACACAGG |
| C8573 | AATTGGAGCTCCACCGCG |
| sgRNA R | CGGAGTCTATCTGATACGTGCAACAGTATACCCTGCCGGTG |
| sgRNA F | CACGTATCAGATAGACTCCGGTTTTAGAGCTAGAAATAGCAAGTT |
| C8574 | GGGAACAAAAGCTGGGTACC |
| SIR2 + HYG^R^ F | TTAATTGGCGTATACCTCGACATTCCCGCTACCTTGAACTCGTTAGCTTAGTAAAACGACGGCCAGTGC |
| SIR2 + HYG^R^ R | TAGTGCCACACGAGAAGAGAATAATTTTTTGAAATGGGCTTGAAACAAGTCACAGGAAACAGCTATGAC |
| SIR2 F | ATGTCAAAACCAGATCTGTC |
| SIR2 R | TCATGAACTTGCATTCCCAA |
| SIR2 F | TGCAGGCCAAAACGATGAAA |
| SIR2 R | GGAGAGGATGGTACGACTGG |
| HST2 F | CTCCCAGATCGGTTCTTCAA |
| HST2 R | ACTGTCCAGACCGGAAAATG |
| HST3 F | GCGTAAAGAGGGTCGTCAAG |
| HST3 R | AGAGGGGGAAGCTTTCTTTG |
| HST4 F | GCTAAAGCGACTGGTCAAGG |
| HST4 R | TGGCTTCACTCTCCTCCACT |
| HST5 F | AGGGACGAGTATCAGGAGCA |
| HST5 R | ATCACCATCAGGGTTCGTTC |
| TSC2 F | CTCGACTTTTCGAGGACCAG |
| TSC2 R | GAAAAGCGCAAGTGATCTCC |
| RHEB F | TCTTCTCGGGTTGAGGATTG |
| RHEB R | GAAAGTGTGCCCCTTGTCAT |
| TOR1 F | TTCAGCCTGTCAGATGTTCG |
| TOR1 R | GACTTGGAAGAAGCGGTGAG |
| LST8 F | GATTCGGGTGGATTACAACG |
| LST8 R | ACGGAACAGTCAAGGTTTGG |
| KOG1 F | GAGCGACTGGATAGGTTTCG |
| KOG1 R | CGGTGGAAGCAGTATTTCGT |
| TTIL F | CTGCAGCCTCAACTACCACA |
| TTIL R | CTGCCACTCGATCTCAACAA |
| TEL2 F | CTATTGTCACCGCAAGCTCA |
| TEL2 R | CTTCATCCTTCCCCTCATCA |
| CKAI1 F | GTCCGCTTCTCGTGATCAAT |
| CKAI1 R | CACCGAATTCAAGACGCTTT |
| AVO1 F | ATCGCCATTGTCTTCTCAC |
| AVO1 R | GCGACTTCATTCGGAATTGT |
| AVO2 F | ATTGTTGTGATGCTGGTGGA |
| AVO2 R | GCTAAAGATCGGCAAGATGC |
| AVO3 F | AAATTCGTCAACACCGAAGG |
| AVO3 R | AGCCGAAACGTTCGTCTCTA |
| SCH9 F | TTTTGCAGATGCCAGAACAG |
| SCH9 R | GCACTTTCGGTCGAGTCTTC |
| TAP42 F | TGACGACCGAGTCAGAGATG |
| TAP42 R | CGCTGTCTTCTATGGCTTCC |
| SIT4 F | GAACGCAGAGGATTGAGGAG |
| SIT4 R | GCAATCCTTCGGTATGGAAA |
| YPK2 F | AGTTGGGCCGAGTAGAACCT |
| YPK2 R | ACACCTTGGCAGAACGTACC |
| GPA1 F | TTCCTGCGAACATCCGTCAT |
| GPA1 R | GACGTATCAGTCGCTTGGGT |
| GIB2 F | GGCAAGCACCTCTACTCTCT |
| GIB2 R | AAAGTCAGGTTGGAGGTCGT |
| GPG1 F | ATGTCCATACGCACAACAAA |
| GPG1 R | CGGAACAGCAGACAGCTTGT |
| GPG2 F | CATTCGCCCCCACAAAACGT |
| GPG2 R | TGGTGCAGCACCCAGTATCT |
| ACA1 F | CCGCCGTATCCTCTTTCTCT |
| ACA1 R | ATGCTACTGGACTTGCTGGT |
| CAN2 F | ACCAACCTCTTCCGACTGAG |
| CAN2 R | TGTTCCCAAGCTCCCTGAAT |
| CAC1 F | AGCAAGTTATCTGGGGCCTT |
| CAC1 R | GCGGCCAATACAGCACTAAA |
| RAS1 F | GAAGCCTTTATTGCCGTCGT |
| RAS1 R | GCATCCTCTACAGCATCCCT |
| RAS2 F | ACGTACGATCCCACCATTGA |
| RAS2 R | GAATCCATTGATCGCGCAGT |
| PKA1 F | CGGGGATCTCACTAAGCGAT |
| PKA1 R | GCACTAACATCCGCCTCTTG |
| PKA2 F | GGTTTGCGGGTGTAGATTGG |
| PKA2 R | CCCTCTCGCTCTCTCCTCTA |
| SOD1 F | AACACCAACGGCTGTACCTC |
| SOD1 R | TTCGACCAATGATGGAGTGA |
| SOD2 F | TTCCCACCTCTGGTGTCTTC |
| SOD2 R | TAGAAAGCGTGCTCCCAGAT |
| AFR1 F | CTTTCCGAGCTGGTGAACTC |
| AFR1 R | CACCTTCGATCACACCAATG |
| AFR2 F | GGTTCCGACTACATGGCTGT |
| AFR2 R | GAGTTCACCAGCTCGGAAAG |
| MDR1 F | CTCTTGATCACATCGCGAAA |
| MDR1 R | ACCGACAATCTTGCTCTGCT |
| ACT1 F | CCCACACTGTCCCCATTTAC |
| ACT1 R | AACCACGCTCCATGAGAATC |

Supplemental Data: Sequencing

KN99α

CAAGATTAGACTGTGCCTGGATAATTCATGGCTTTGAATCTAGCTTCGCAGTTCGTTTCTCTTTACTCTCGCTTCATCTATCATTCCAACGGTCTTGGCTTTAATTGGCGTATACCTCGACATTCCCGCTACCTTGAACTCGTTAGCTTAATGTCAAAACCAGATCTGTCGCAATATATCATGAGTGAAGAAGTTCCCCTGCCACCAGCGCAAGAGATCAGGCCCGGAGACACGTATCAGATAGACTCCGAGGAATACGATTCCGATCAAGAACGTGATAATTTGACTGATGGAGAATATTATAGACTAGTTGAAGAAGGTGGGTTTGGTTTCTCATGTGTTTGAGCTAGGACACTTCTCTGGTGTTTGTAGTCCATACAAATGTGCTGCATGGCGGTGCTGACAAATGCTGCCTGTAGCGGAGAATGGATACACTGATGAAGAGATGGATCAATATACCCGCCAGCTCAAGGAGGATGGCATCGTAAACTTCTTGAGAAGCTATCTAACATTAACTGAGGACGGTGAGATGCGATCGCTTCGAAAATTATTATTAGGCTTTGGTCTTGTTCCTGTAGGTCGCTGTCGTTCCGTGGTTTCAATCAGAAGGGCAAACTCATCGCCGAACAGCCTCTTTCTCTTCGGTCTCCGGAAACTCCCAACCTTCAATTACTCCCATTTGCCAAAGTTGCCCTTTCTAAAATTTTACGCAGACGTGAACGTCTTCGAGACCTCTCGTCTTTAGATGATGCTATCTCCTTACTGGCCAAGTCTAAGAAGATCATTGTGCTCTCTGGGGCAGAATCTCGACTTCTTGCGGTATACCCGACTTCCGTTCAAGTACTGGTCTGTACGCCCAACTGCAGGAAGAGGGAAAATATGAGCTGGACGATCCGCAGCAGATGTTTGACATCAGATTCTTCAGAGAGAAGCCTGAAGTTTTCTAGTGTGTGTCGTTTATAGCTAACATTGTATATATCGTTGACGTCCTTTGGATAGCTCCTTCGCCAAGCAAATCTATCCCAGTAATTTTGTTCCCAGTCCTTGTCATAGATGGATCAAGATGTTAGAGGATCGAGGTGTTGTAAGCTTATTTTGATACGTCCTTGAATTTAACATTCTTATTTTCTACAGCTTTTGAGGAACTATACACAAAACATAGATACCTTGGAAAGCCTGGCAGGCGTGGAGAGAGTTTTGCAATGCCACGGTTCCTTCAAGTCTGCTTCCTGCCTTGTAAGCTCAAATTTCATTTAAATTCGTCATCCATTACTTGCTGATTATCACTATCGCTAGCGTTGTAAACACCGGGTGCCAGGCCGGACTATAGAACCGTACATAATGTCCCAGCAAATCCCTTACTGTGGTTCTTGCCGTGAAATATGTGCTGCTGAGCGAGAGGCAAGAAGAGCCTATAGAGAAAAATTAAAAAAGTTGAAAGCCAAAGCTAAAGGGAAGAGCAAAGCGGATGAGTGGGATGATGGCGACGACGATGAAGATGATGAAAGTGCGGATGAATGGGGAGGAGGTGAACCCGGTATCATCAAGGTGAGTAGAATATTCTGCGATAAAATGAAATGCTCTGGCCAACACATCATACAGCCTGATATAACCTTTTTCGGCCAGGCCCTCGATTCAGAATTTGATGAATGTCTATTTAAAGACCGCGAAGAGGTGGACTTGCTTGTTGTTATAGGTACCAGTTTGAAGGTGGCGCCAGTGAGCGAAGTACTCAGTGAGTTGTTTGTTTTTTTTTACACTTTAGTTAGTGACTAAGATGCTAAACTAAACCAACTTTTTTTTTCTCAGCACACATACCACATTCTGTACCACAAATATTCATCAATCTCACTCCTGTGTACCATGTACAACCAGACGTAAGTGTTTTTTTCCACCTATTCTTCAACCTTCTCGACAGTTGTTTACCGTTTTGTGTCTGTCTGAAGATATCTCTCCTGGGCGATGCCGACAGCATAGTGACCTATCTTTCCGATAGGCTAGGCTGGCCTATCCCTCCCCCGACAGTACTTCCGACACCACCATCTGCCATTGGCGAGGGCAAGAGCGAGGAGATAGCTTCTGCAGGCCAAAACGATGAAATCAAGATGGAGCGGGCTACGCAGGTGGTGATTCCCCCTGAAGAAGCACAGTGGCTCACCGTCGATGAAGAGTAAGTGTCCCTTTTTTTTTTTTTGGCAAATTGGACGTAATATGGGTGTAAAGAGGGGGCTAACTTTGTGGTCAGACGCCATTTCCACCTTCTCCGCCGAAAGGGTGATCCCGCCATCCAGGCTGAGACTACCGTCACTGCTCCAGTCGTACCATCCTCTCCAAAGCCCGAACGTTTTCCCCAGCCTCAACCTCAATCCACTGGAAACACGACAGAACAACCGTCATCTCCACCTCCGGTCCCTATGCAAGTAGGCGAGGCAGAGGAAGGCTACGTCTCTGATGAAGAAGATGAAGCTGATGAACCGCCTAGAAAACGATCAAAGGTGGTGGATAGTTTGCTTGGGAATGCAAGTTCATGAGGACTTGTTTCAAGCCCATTTCAAAAAATTATTCTCTTCTCGTGTGGCACTATCGTTGGGGTTGCATGGGTGTAGCTAACGATAATATTGTCAAATGTTGTTTGGAGAAGCCGATGTTGATTTAGACAGCGCTCCAATGTCGCCGCCTCTAT

Legend:

5’ UTR and 3’ UTR

Primer Forward and Reverse

ATG - start of *SIR2* gene

KN99α *Δsir2 -*1

CAAGATTAGACTGTGCCTGGATAATTCATGGCTTTGAATCTAGCTTCGCAGTTCGTTTCTCTTTACTCTCGCTTCATCTATCATTCCAACGGTCTTGGCTTTAATTGGCGTATACCTCGACATTCCCGCTACCTTGAACTCGTTAGCTTAGTTGGATCCGCTGCTAGGCGCGCCGTGAGCTGCGAGGATGTGAGCTGGAGAGCGGCGCACGGGAAGGGGAGGACTCACATAAGCATGCAGGATTCGAGTGGCATGGTGTGCGCTGAGTGTATGGTTGTCGGAGGAGAGGATGATGGTAACAACAATAGCAGCAACGTCACTCGACGCGCGTCCGGTGTGCCACACGGGGTAACGCCGAGTCGCCGTCAGGGTCGCCGAGACCACTCTCACAGCGTCACCGTTGGCACCAGCTCAGCTTACAGCTTCTATCCTCCGCCAGCATCCACATACATCCCCTATACCGCATCCCCCACCCACTGCCCAAGGTGAGTCATCTTCCCGCCCCCTTCCCTTGCCCGCCACTCAGTCCTCCATCCTCCACTAATCCACCTTATCGCACCCACCGCCTATCGCACATCCGAGCACAATGCTGGGCCTGCCAGGGGCTGCTAGATGGTGCTCTCCCCACGCTGATCTGCATGCCGGCCATTGGATCATGGGTGCTAGGTGCTGGGTGCTGGATGTTGGATGCTGGATGCTGGGTGCACGCTTGGTCATTTCCTTCCAGGATTGACGGTCGCCGAGAGGACGACGTGGCGTTCGACAACGAGGGCCGATAGCACCGCATCGCCTCGACCTGCATCCATCTCGCCTTGTCCTTTTGGTGCAACAATCCATCCGTGCTGGTGCCACACGCATAGCTGGAAGAGATGGATGTGCGTTGAACAGAGCTGCCGTCAGGACTTTTTGGTGCACGGACCCTATTGTCCTCCCCAATCTTCACCGCGTCTCCTAATATGCAGCCTCTTTGCTAATTGTCTTTTTCCATTAGTAAACTCGCCCAACATGTCTATGGCGGCCGCCACTCTTGACGACACGGCTTACCGGTACCGCACCAGTGTCCCGGGGGACGCCGAGGCCATCGAGGCACTGGATGGGTCCTTCACCACCGACACCGTCTTCCGCGTCACCGCCACCGGGGACGGCTTCACCCTGCGGGAGGTGCCGGTGGACCCGCCCCTGACCAAGGTGTTCCCCGACGACGAATCGGACGACGAATCGGACGACGGGGAGGACGGCGACCCGGACTCCCGGACGTTCGTCGCGTACGGGGACGACGGCGACCTGGCGGGCTTCGTGGTCGTCTCGTACTCCGGCTGGAACCGCCGGCTGACCGTCGAGGACATCGAGGTCGCCCCGGAGCACCGGGGGCACGGGGTCGGGCGCGCGTTGATGGGGCTCGCGACGGAGTTCGCCCGCGAGCGGGGCGCCGGGCACCTCTGGCTGGAGGTCACCAACGTCAACGCACCGGCGATCCACGCGTACCGGCGGATGGGGTTCACCCTCTGCGGCCTGGACACCGCCCTGTACGACGGCACCGCCTCGGACGGCGAGCAGGCGCTCTACATGAGCATGCCCTGCCCCTAAGAATTCGTGAAGGCGGTAAGGGGTTAATTTTCCTTAGAGGGTGTATATATACATATTAGAGAAGTGATACAATTTTAGCACACTGCGAATCCGAGACAGACATCGTGTCAATCATCTTTTTTGACATTATATGCCCATTAATCTATCTACAGACAACAATACCATCCTTCCCACCCTCAGCAACGCCGTTGAATCCTCAGGATCTTCATGGCTCCTTGTCTCTGAAACCAGGGTCAGCGGCCGCATCCCTGCATCCAACGGACTTGTTTCAAGCCCATTTCAAAAAATTATTCTCTTCTCGTGTGGCACTATCGTTGGGGTTGCATGGGTGTAGCTAACGATAATATTGTCAAATGTTGTTTGGAGAAGCCGATGTTGATTTAGACAGCGCTCCAATGTCGCCGCCTCTATATAGGGAGGGGCCATGTAATCATTTAAAATCATTTAAACATTTTCCCACAGACTGGGCGAGCAGGCGC

Legend:

5’ UTR and 3’ UTR

NAT^R^ cassette

KN99α *Δsir2* -2

CAAGATTAGACTGTGCCTGGATAATTCATGGCTTTGAATCTAGCTTCGCAGTTCGTTTCTCTTTACTCTCGCTTCATCTATCATTCCAACGGTCTTGGCTTTAATTGGCGTATACCTCGACATTCCCGCTACCTTGAACTCGTTAGCTTAGTAAAACGACGGCCAGTGCCAAGCTTGGTACCGAGCTCGGATCCACTAGTAACGGCCGCCAGTGTGCTGGAATTCGCCCTTGGTTTATCTGTATTAACACGGAAGAGATGTAGAAACTAGCTTCCTGGTTTCAGAGACAAGGAGCCATGAAGATCCTGAGGATTCAACGGCGTTGCTGAGGGTGGGAAGGATGGTATTGTTGTCTGTAGATAGATTAATGGGCATATAATGTCAAAAAAGATGATTGACACGATGTCTGTCTCGAATTCGCAGTGTGCTAAAATTGTATCACTTCTCTAATATGTATATATACACCCTCTAAGGAAAATTAACCCCTTACCGCCTTCACCTATTCCTTTGCCCTCGGACGAGTGCTGGGGCGCCGGTTTCCACTATCGGCGAGTACTTCTACACAGCCATCGGTCCAGACGGCCGCGCTTCTGCGGGCGATTTGTGTACGCCCGACAGTCCCGGCTCCGGATCGGACGATTGCGTCGCATCGACCCTGCGCCCAAGCTGCATCATCGAAATTGCCGTCAACCAAGCTCTGATAGAGTTGGTCAAGACCAATGCGGAGCATATACGCCCGGAGCCGCGGCGATCCTGCAAGCTCCGGATGCCTCCGCTCGAAGTAGCGCGTTTGCTGCTCCATACAAGCCAACCACGGCCTYCAGAAGAAGATGTTGGCGACCTCGTATTGGGAATCCCCGAACATCGCCTCGCTCCAGTCAATGACCGCTGTTATGCGGCCATTGTCCGTCAGGACATTGTTGGAGCCGAAATCCGCGTGCACGAGGTGCCGGACTTCGGGGCAGTCCTCGGCCCAAAGCATCAGCTCATCGAGAGCCTGCGCGACGGACGCACTGACGGTGTCGTCCATCACAGTTTGCCAGTGATACACATGGGGATCAGCAATCGCGCATATGAAATCACGCCATGTAGTGTATTGACCGATTCCTTGCGGTCCGAATGGGCCGAACCCGCTCGTCTGGCTAAGATCGGCCGCAGCGATCGCATCCATGGCCTCCGCGACCGGCTGCAGAACAGCGGGCAGTTCGGTTTCAGGCAGGTCTTGCAACGTGACACCCTGTGCACGGCGGGAGATGCAATAGGTCAGGCTCTCGCTGAATTCCCCAATGTCAAGCACTTCCGGAATCGGGAGCGCGGCCGATGCAAAGTGCCGATAAACATAACGATCTTTGTAGAAACCATCGGCGCAGCTATTTACCCGCAGGACATATCCACGCCCTCCTACATCGAAGCTGAAAGCACGAGATTCTTCGCCCTCCGAGAGCTGCATCAGGTCGGAGACGCTGTCGAACTTTTCGATCAGAAACTTCTCGACAGACGTCGCGGTGAGTTCAGGCTTTTTCATAGACATGTTGGGCGAGTTTACTAATGGAAAAAGACAATTAGCAAAGAGGCTGCATATCAGAGACGCGGTGAAGATTGGGGAGGACAATAGGGTCCGTGCACCAAAAAGTCCTGACGGCAGCTCTGTTCAACGCACATCCATCTCTTCCAGCTATGCGTGTGGCACCAGCACGGATGGATTGTTGCACCAAAAGGACAAGGCGAGATGGATGCAGGTCGAGGCGATGCGGTGCTATCGGCCCTCGTTGTCGAACGCCACGTCGTCCTCTCGGCGACCGTCAATCCTGGAAGGAAATGACCAAGCGTGCACCCAGCATCCAGCATCCAACATCCAGCACCCAGCACCTAGCACCCATGATCCAATGGCCGGCATGCAGATCAGCGTGGGGAGAGCACCATCTAGCAGCCCCTGGCAGGCCCAGCATTGTGCTCGGATGTGCGATAGGCGGTGGGTGCGATAAGGTGGATTAGTGGAGGATGGAGGACTGAGTGGCGGGCAAGGGAAGGGGGCGGGAAGATGACTCACCTTGGGCAGTGGGTGGGGGATGCGGTATAGGGGATGTATGTGGATGCTGGCGGAGGATAGAAGCTGTAAGCTGAGCTGGTGCCAACGGTGACGCTGTGAGAGTGGTCTCGGCGACCCTGACGGCGACTCGGCGTTACCCCGTGTGGCACACCGGACGCGCGTCGAGTGACGTTGCTGCTATTGTTGTTACCATCATCCTCTCCTCCGACAACCATACACTCAGTGCACACCATGCCACTCGAATCCTGCATGCTTATGTGAGTCCTCCCCTTCCCGTGCGCCGCTCTCCAGCTCACATCCTCGCAGCAAGGGCGAATTCTGCAGATATCCATCACACTGGCGGCCGCTCGAGCATGCATCTAGAGGATCCCCGGGTACCGAGCTCGATATCTTAATTAAGAATTCGTAATCATGTCATAGCTGTTTCCTGTGACTTGTTTCAAGCCCATTTCAAAAAATTATTCTCTTCTCGTGTGGCACTATCGTTGGGGTTGCATGGGTGTAGCTAACGATAATATTGTCAAATGTTGTTTGGAGAAGCCGATGTTGATTTAGACAGCGCTCCAATGTCGCCGCCTCTAT

Legend:

5’ UTR and 3’ UTR

Primer Forward and Reverse

HYG^R^ cassette

KN99**a**

CAAGATTAGACTGTGCCTGGATAATTCATGGCTTTGAATCTAGCTTCGCAGTTCGTTTCTCTTTACTCTCGCTTCATCTATCATTCCAACGGTCTTGGCTTTAATTGGCGTATACCTCGACATTCCCGCTACCTTGAACTCGTTAGCTTAATGTCAAAACCAGATCTGTCGCAATATATCATGAGTGAAGAAGTTCCCCTGCCACCAGCGCAAGAGATCAGGCCCGGAGACACGTATCAGATAGACTCCGAGGAATACGATTCCGATCAAGAACGTGATAATTTGACTGATGGAGAATATTATAGACTAGTTGAAGAAGGTGGGTTTGGTTTCTCATGTGTTTGAGCTAGGACACTTCTCTGGTGTTTGTAGTCCATACAAATGTGCTGCATGGCGGTGCTGACAAATGCTGCCTGTAGCGGAGAATGGATACACTGATGAAGAGATGGATCAATATACCCGCCAGCTCAAGGAGGATGGCATCGTAAACTTCTTGAGAAGCTATCTAACATTAACTGAGGACGGTGAGATGCGATCGCTTCGAAAATTATTATTAGGCTTTGGTCTTGTTCCTGTAGGTCGCTGTCGTTCCGTGGTTTCAATCAGAAGGGCAAACTCATCGCCGAACAGCCTCTTTCTCTTCGGTCTCCGGAAACTCCCAACCTTCAATTACTCCCATTTGCCAAAGTTGCCCTTTCTAAAATTTTACGCAGACGTGAACGTCTTCGAGACCTCTCGTCTTTAGATGATGCTATCTCCTTACTGGCCAAGTCTAAGAAGATCATTGTGCTCTCTGGGGCAGGAATCTCGACTTCTTGCGGTATACCCGACTTCCGTTCAAGTACTGGTCTGTACGCCCAACTGCAGGAAGAGGGAAAATATGAGCTGGACGATCCGCAGCAGATGTTTGACATCAGATTCTTCAGAGAGAAGCCTGAAGTTTTCTAGTGTGTGTCGTTTATAGCTAACATTGTATATATCGTTGACGTCCTTTGGATAGCTCCTTCGCCAAGCAAATCTATCCCAGTAATTTTGTTCCCAGTCCTTGTCATAGATGGATCAAGATGTTAGAGGATCGAGGTGTTGTAAGCTTATTTTGATACGTCCTTGAATTTAACATTCTTATTTTCTACAGCTTTTGAGGAACTATACACAAAACATAGATACCTTGGAAAGCCTGGCAGGCGTGGAGAGAGTTTTGCAATGCCACGGTTCCTTCAAGTCTGCTTCCTGCCTTGTAAGCTCAAATTTCATTTAAATTCGTCATCCATTACTTGCTGATTATCACTATCGCTAGCGTTGTAAACACCGGGTGCCAGGCCGGACTATAGAACCGTACATAATGTCCCAGCAAATCCCTTACTGTGGTTCTTGCCGTGAAATATGTGCTGCTGAGCGAGAGGCAAGAAGAGCCTATAGAGAAAAATTAAAAAAGTTGAAAGCCAAAGCTAAAGGGAAGAGCAAAGCGGATGAGTGGGATGATGGCGACGACGATGAAGATGATGAAAGTGCGGATGAATGGGGAGGAGGTGAACCCGGTATCATCAAGGTGAGTAGAATATTCTGCGATAAAATGAAATGCTCTGGCCAACACATCATACAGCCTGATATAACCTTTTTCGGCCAGGCCCTCGATTCAGAATTTGATGAATGTCTATTTAAAGACCGCGAAGAGGTGGACTTGCTTGTTGTTATAGGTACCAGTTTGAAGGTGGCGCCAGTGAGCGAAGTACTCAGTGAGTTGTTTGTTTTTTTTTACACTTTAGTTAGTGACTAAGATGCTAAACTAAACCAACTTTTTTTTTCTCAGCACACATACCACATTCTGTACCACAAATATTCATCAATCTCACTCCTGTGTACCATGTACAACCAGACGTAAGTGTTTTTTTCCACCTATTCTTCAACCTTCTCGACAGTTGTTTACCGTTTTGTGTCTGTCTGAAGATATCTCTCCTGGGCGATGCCGACAGCATAGTGACCTATCTTTCCCATAGGCTAGGCTGGCCTATCCCTCCCCCGACAGTACTTCCGACACCMCCATCTGCCATTGGCGAGGGCAAGAGCGAGGAGATAGCTTCTGCAGGCCAAAACGATGAAATCAAGATGGAGCGGGCTACGCAGGTGGTGATTCCCCCTGAAGAAGCACAGTGGCTCACCGTCGATGAAGAGTAAGTGTCCCTTTTTTTTTTTTTGGCAAATTGGACGTAATATGGGTGTAAAGAGGGGGCTAACTTTGTGGTCAGACGCCATTTCCACCTTCTCCGCCGAAAGGGTGATCCCGCCATCCAGGCTGAGACTACCGTCACTGCTCCAGTCGTACCATCCTCTCCAAAGCCCGAACGTTTTCCCCAGCCTCAACCTCAATCCACTGGAAACACGACAGAACAACCGTCATCTCCACCTCCGGTCCCTATGCAAGTAGGCGAGGCAGAGGAAGGCTACGTCTCTGATGAAGAAGATGAAGCTGATGAACCGCCTAGAAAACGATCAAAGGTGGTGGATAGTTTGCTTGGGAATGCAAGTTCATGAGGACTTGTTTCAAGCCCATTTCAAAAAATTATTCTCTTCTCGTGTGGCACTATCGTTGGGGTTGCATGGGTGTAGCTAACGATAATATTGTCAAATGTTGTTTGGAGAAGCCGATGTTGATTTAGACAGCGCTCCAATGTCGCCGCCTCTAT

Legend:

5’ UTR and 3’ UTR

Primer Forward and Reverse

ATG - start of *SIR2* gene

KN99**a** *Δsir2* -1

CAAGATTAGACTGTGCCTGGATAATTCATGGCTTTGAATCTAGCTTCGCAGTTCGTTTCTCTTTACTCTCGCTTCATCTATCATTCCAACGGTCTTGGCTTTAATTGGCGTATACCTCGACATTCCCGCTACCTTGAACTCGTTAGCTTAGTAAAACGACGGCCAGTGCCAAGCTTGGTACCGAGCTCGGATCCACTAGTAACGGCCGCCAGTGTGCTGGAATTCGCCCTTGGTTTATCTGTATTAACACGGAAGAGATGTAGAAACTAGCTTCCTGGTTTCAGAGACAAGGAGCCATGAAGATCCTGAGGATTCAACGGCGTTGCTGAGGGTGGGAAGGATGGTATTGTTGTCTGTAGATAGATTAATGGGCATATAATGTCAAAAAAGATGATTGACACGATGTCTGTCTCGAATTCGCAGTGTGCTAAAATTGTATCACTTCTCTAATATGTATATATACACCCTCTAAGGAAAATTAACCCCTTACCGCCTTCACCTATTCCTTTGCCCTCGGACGAGTGCTGGGGCGCCGGTTTCCACTATCGGCGAGTACTTCTACACAGCCATCGGTCCAGACGGCCGCGCTTCTGCGGGCGATTTGTGTACGCCCGACAGTCCCGGCTCCGGATCGGACGATTGCGTCGCATCGACCCTGCGCCCAAGCTGCATCATCGAAATTGCCGTCAACCAAGCTCTGATAGAGTTGGTCAAGACCAATGCGGAGCATATACGCCCGGAGCCGCGGCGATCCTGCAAGCTCCGGATGCCTCCGCTCGAAGTAGCGCGTTTGCTGCTCCATACAAGCCAACCACGGCCTCCAGAAGAAGATGTTGGCGACCTCGTATTGGGAATCCCCGAACATCGCCTCGCTCCAGTCAATGACCGCTGTTATGCGGCCATTGTCCGTCAGGACATTGTTGGAGCCGAAATCCGCGTGCACGAGGTGCCGGACTTCGGGGCAGTCCTCGGCCCAAAGCATCAGCTCATCGAGAGCCTGCGCGACGGACGCACTGACGGTGTCGTCCATCACAGTTTGCCAGTGATACACATGGGGATCAGCAATCGCGCATATGAAATCACGCCATGTAGTGTATTGACCGATTCCTTGCGGTCCGAATGGGCCGAACCCGCTCGTCTGGCTAAGATCGGCCGCAGCGATCGCATCCATGGCCTCCGCGACCGGCTGCAGAACAGCGGGCAGTTCGGTTTCAGGCAGGTCTTGCAACGTGACACCCTGTGCACGGCGGGAGATGCAATAGGTCAGGCTCTCGCTGAATTCCCCAATGTCAAGCACTTCCGGAATCGGGAGCGCGGCCGATGCAAAGTGCCGATAAACATAACGATCTTTGTAGAAACCATCGGCGCAGCTATTTACCCGCAGGACATATCCACGCCCTCCTACATCGAAGCTGAAAGCACGAGATTCTTCGCCCTCCGAGAGCTGCATCAGGTCGGAGACGCTGTCGAACTTTTCGATCAGAAACTTCTCGACAGACGTCGCGGTGAGTTCAGGCTTTTTCATAGACATGTTGGGCGAGTTTACTAATGGAAAAAGACAATTAGCAAAGAGGCTGCATATCAGAGACGCGGTGAAGATTGGGGAGGACAATAGGGTCCGTGCACCAAAAAGTCCTGACGGCAGCTCTGTTCAACGCACATCCATCTCTTCCAGCTATGCGTGTGGCACCAGCACGGATGGATTGTTGCACCAAAAGGACAAGGCGAGATGGATGCAGGTCGAGGCGATGCGGTGCTATCGGCCCTCGTTGTCGAACGCCACGTCGTCCTCTCGGCGACCGTCAATCCTGGAAGGAAATGACCAAGCGTGCACCCAGCATCCAGCATCCAACATCCAGCACCCAGCACCTAGCACCCATGATCCAATGGCCGGCATGCAGATCAGCGTGGGGAGAGCACCATCTAGCAGCCCCTGGCAGGCCCAGCATTGTGCTCGGATGTGCGATAGGCGGTGGGTGCGATAAGGTGGATTAGTGGAGGATGGAGGACTGAGTGGCGGGCAAGGGAAGGGGGCGGGAAGATGACTCACCTTGGGCAGTGGGTGGGGGATGCGGTATAGGGGATGTATGTGGATGCTGGCGGAGGATAGAAGCTGTAAGCTGAGCTGGTGCCAACGGTGACGCTGTGAGAGTGGTCTCGGCGACCCTGACGGCGACTCGGCGTTACCCCGTGTGGCACACCGGACGCGCGTCGAGTGACGTTGCTGCTATTGTTGTTACCATCATCCTCTCCTCCGACAACCATACACTCAGTGCACACCATGCCACTCGAATCCTGCATGCTTATGTGAGTCCTCCCCTTCCCGTGCGCCGCTCTCCAGCTCACATCCTCGCAGCAAGGGCGAATTCTGCAGATATCCATCACACTGGCGGCCGCTCGAGCATGCATCTAGAGGATCCCCGGGTACCGAGCTCGATATCTTAATTAAGAATTCGTAATCATGTCATAGCTGTTTCCTGTGACTTGTTTCAAGCCCATTTCAAAAAATTATTCTCTTCTCGTGTGGCACTATCGTTGGGGTTGCATGGGTGTAGCTAACGATAATATTGTCAAATGTTGTTTGGAGAAGCCGATGTTGATTTAGACAGCGCTCCAATGTCGCCGCCTCTAT

Legend:

5’ UTR and 3’ UTR

Primer Forward and Reverse

HYG^R^ cassette

KN99**a** *Δsir2* -2

CAAGATTAGACTGTGCCTGGATAATTCATGGCTTTGAATCTAGCTTCGCAGTTCGTTTCTCTTTACTCTCGCTTCATCTATCATTCCAACGGTCTTGGCTTTAATTGGCGTATACCTCGACATTCCCGCTACCTTGAACTCGTTAGCTTAGTAAAACGACGGCCAGTGCCAAGCTTGGTACCGAGCTCGGATCCACTAGTAACGGCCGCCAGTGTGCTGGAATTCGCCCTTGGTTTATCTGTATTAACACGGAAGAGATGTAGAAACTAGCTTCCTGGTTTCAGAGACAAGGAGCCATGAAGATCCTGAGGATTCAACGGCGTTGCTGAGGGTGGGAAGGATGGTATTGTTGTCTGTAGATAGATTAATGGGCATATAATGTCAAAAAAGATGATTGACACGATGTCTGTCTCGAATTCGCAGTGTGCTAAAATTGTATCACTTCTCTAATATGTATATATACACCCTCTAAGGAAAATTAACCCCTTACCGCCTTCACCTATTCCTTTGCCCTCGGACGAGTGCTGGGGCGTCGGTTTCCACTATCGGCGAGTACTTCTACACAGCCATCGGTCCAGACGGCCGCGCTTCTGCGGGCGATTTGTGTACGCCCGACAGTCCCGGCTCCGGATCGGACGATTGCGTCGCATCGACCCTGCGCCCAAGCTGCATCATCGAAATTGCCGTCAACCAAGCTCTGATAGAGTTGGTCAAGACCAATGCGGAGCATATACGCCCGGAGCCGCGGCGATCCTGCAAGCTCCGGATGCCTCCGCTCGAAGTAGCGCGTCTGCTGCTCCATACAAGCCAACCACGGCCTCCAGAAGAAGATGTTGGCGACCTCGTATTGGGAATCCCCGAACATCGCCTCGCTCCAGTCAATGACCGCTGTTATGCGGCCATTGTCCGTCAGGACATTGTTGGAGCCGAAATCCGCGTGCACGAGGTGCCGGACTTCGGGGCAGTCCTCGGCCCAAGGCATCAGCTCATCGAGAGCCTGCGCGACGGACGCACTGACGGTGTCGTCCATCACAGTTTGCCAGTGATACACATGGGGATCAGCAATCGCGCATATGAAATCACGCCATGTAGTGTATTGACCGATTCCTTGCGGTCCGAATGGGCCGAACCCGCTCGTCTGGCTAAGATCGGCCGCAGCGATCGCATCCATGGCCTCCGCGACCGGCTGCAGAACAGCGGGCAGTTCGGTTTCAGGCAGGTCTTGCAACGTGACACCCTGTGCACGGCGGGAGATGCAATAGGTCAGGCTCTCGCTGAATTCCCCAACGTCAAGCACTTCCGGAATCGGGAGCGCGGCCGATGCAAAGTGCCGATAAACATAACGATCTTTGTAGAAACCATCGGCGCAGCTATTTACCCGCAGGACATATCCACGCCCTCCTACATCGAAGCTGAAAGCACGAGATTCTTCGCCCTCCGAGAGCTGCATCAGGTCGGAGACGCTGTCGAACTTTTCGATCAGAAACTTCTCGACAGACGTCGCGGTGAGTTCAGGCTTTTTCATAGACATGTTGGGCGAGTTTACTAATGGAAAAAGACAATTAGCAAAGAGGCTGCATATTAGGAGACGCGGTGAAGATTGGGGAGGACAATAGGGTCCGTGCACCAAAAAGTCCTGACGGCAGCTCTGTTCAACGCACATCCATCTCTTCCAGCTATGCGTGTGGCACCAGCACGGATGGATTGTTGCACCAAAAGGACAAGGCGAGATGGATGCAGGTCGAGGCGATGCGGTGCTATCGGCCCTCGTTGTCGAACGCCACGTCGTCCTCTCGGCGACCGTCAATCCTGGAAGGAAATGACCAAGCGTGCACCCAGCATCCAGCATCCAACATCCAGCACCCAGCACCTAGCACCCATGATCCAATGGCCGGCATGCAGATCAGCGTGGGGAGAGCACCATCTAGCAGCCCCTGGCAGGCCCAGCATTGTGCTCGGATGTGCGATAGGCGGTGGGTGCGATAAGGTGGATTAGTGGAGGATGGAGGACTGAGTGGCGGGCAAGGGAAGGGGGCGGGAAGATGACTCACCTTGGGCAGTGGGTGGGGGATGCGGTATAGGGGATGTATGTGGATGCTGGCGGAGGATAGAAGCTGTAAGCTGAGCTGGTGCCAACGGTGACGCTGTGAGAGTGGTCTCGGCGACCCTGACGGCGACTCGGCGTTACCCCGTGTGGCACACCGGACGCGCGTCGAGTGACGTTGCTGCTATTGTTGTTACCATCATCCTCTCCTCCGACAACCATACACTCAGTGCACACCATGCCACTCGAATCCTGCATGCTTATGTGAGTCCTCCCCTTCCCGTGCGCCGCTCTCCAGCTCACATCCTCGCAGCAAGGGCGAATTCTGCAGATATCCATCACACTGGCGGCCGCTCGAGCATGCATCTAGAGGATCCCCGGGTACCGAGCTCGATATCTTAATTAAGAATTCGTAATCATGTCATAGCTGTTTCCTGTGACTTGTTTCAAGCCCATTTCAAAAAATTATTCTCTTCTCGTGTGGCACTATCGTTGGGGTTGCATGGGTGTAGCTAACGATAATATTGTCAAATGTTGTTTGGAGAAGCCGATGTTGATTTAGACAGCGCTCCAATGTCGCCGCCTCTAT

Legend:

5’ UTR and 3’ UTR

Primer Forward and Reverse

HYG^R^ cassette
